# Genetic topography and cortical cell loss in Huntington's disease link development and neurodegeneration

**DOI:** 10.1093/brain/awad275

**Published:** 2023-08-17

**Authors:** Carlos Estevez-Fraga, Andre Altmann, Christopher S Parker, Rachael I Scahill, Beatrice Costa, Zhongbo Chen, Claudia Manzoni, Angeliki Zarkali, Alexandra Durr, Raymund A C Roos, Bernhard Landwehrmeyer, Blair R Leavitt, Geraint Rees, Sarah J Tabrizi, Peter McColgan

**Affiliations:** Department of Neurodegenerative Disease, University College London, London WC1B 5EH, UK; Centre for Medical Image Computing, University College London, London WC1V 6LJ, UK; Centre for Medical Image Computing, University College London, London WC1V 6LJ, UK; Department of Neurodegenerative Disease, University College London, London WC1B 5EH, UK; Department of Neurodegenerative Disease, University College London, London WC1B 5EH, UK; Gladstone Institutes, San Francisco, CA 94158, USA; Department of Neurodegenerative Disease, University College London, London WC1B 5EH, UK; School of Pharmacy, University College London, London WC1N 1AX, UK; Dementia Research Centre, University College London, London WC1N 3AR, UK; Sorbonne Université, Paris Brain Institute (ICM), AP-HP, Inserm, CNRS, Paris 75013, France; Department of Neurology, Leiden University Medical Centre, Leiden 2333, The Netherlands; Department of Neurology, University of Ulm, Ulm 89081, Germany; Centre for Molecular Medicine and Therapeutics, Department of Medical Genetics, University of British Columbia, Vancouver BC V5Z 4H4Canada; Division of Neurology, Department of Medicine, University of British Columbia Hospital, Vancouver BC V6T 2B5, Canada; Wellcome Centre for Human Neuroimaging, UCL Queen Square Institute of Neurology, University College London, London WC1N 3AR, UK; Department of Neurodegenerative Disease, University College London, London WC1B 5EH, UK; Department of Neurodegenerative Disease, University College London, London WC1B 5EH, UK

**Keywords:** Huntington’s disease, atrophy, diffusivity, gene expression, imaging transcriptomics

## Abstract

Cortical cell loss is a core feature of Huntington’s disease (HD), beginning many years before clinical motor diagnosis, during the premanifest stage. However, it is unclear how genetic topography relates to cortical cell loss. Here, we explore the biological processes and cell types underlying this relationship and validate these using cell-specific post-mortem data.

Eighty premanifest participants on average 15 years from disease onset and 71 controls were included. Using volumetric and diffusion MRI we extracted HD-specific whole brain maps where lower grey matter volume and higher grey matter mean diffusivity, relative to controls, were used as proxies of cortical cell loss. These maps were combined with gene expression data from the Allen Human Brain Atlas (AHBA) to investigate the biological processes relating genetic topography and cortical cell loss.

Cortical cell loss was positively correlated with the expression of developmental genes (i.e. higher expression correlated with greater atrophy and increased diffusivity) and negatively correlated with the expression of synaptic and metabolic genes that have been implicated in neurodegeneration. These findings were consistent for diffusion MRI and volumetric HD-specific brain maps.

As wild-type huntingtin is known to play a role in neurodevelopment, we explored the association between wild-type huntingtin (*HTT*) expression and developmental gene expression across the AHBA. Co-expression network analyses in 134 human brains free of neurodegenerative disorders were also performed. *HTT* expression was correlated with the expression of genes involved in neurodevelopment while co-expression network analyses also revealed that *HTT* expression was associated with developmental biological processes.

Expression weighted cell-type enrichment (EWCE) analyses were used to explore which specific cell types were associated with HD cortical cell loss and these associations were validated using cell specific single nucleus RNAseq (snRNAseq) data from post-mortem HD brains.

The developmental transcriptomic profile of cortical cell loss in preHD was enriched in astrocytes and endothelial cells, while the neurodegenerative transcriptomic profile was enriched for neuronal and microglial cells. Astrocyte-specific genes differentially expressed in HD post-mortem brains relative to controls using snRNAseq were enriched in the developmental transcriptomic profile, while neuronal and microglial-specific genes were enriched in the neurodegenerative transcriptomic profile.

Our findings suggest that cortical cell loss in preHD may arise from dual pathological processes, emerging as a consequence of neurodevelopmental changes, at the beginning of life, followed by neurodegeneration in adulthood, targeting areas with reduced expression of synaptic and metabolic genes. These events result in age-related cell death across multiple brain cell types.

## Teaser

Using multimodal MRI, Estevez-Fraga *et al.* reveal associations between cortical cell loss in premanifest Huntington's disease and the expression of developmental genes and neurodegenerative genes. The developmental profile is enriched in astrocytes and endothelial cells and the neurodegenerative profile in neurons and microglia.

See Schultz *et al*. (https://doi.org/10.1093/brain/awad353) for a scientific commentary on this article.

## Introduction

Huntington’s disease (HD) is an adult-onset autosomal dominant neurodegenerative disorder resulting in a triad of cognitive, motor and psychiatric symptoms.^1^ It is caused by cytosine-adenine-guanine (CAG) repeat expansions in the huntingtin (*HTT*) gene, encoding for the toxic mutant huntingtin (mHTT) protein.^2^ Longer CAG repeats are associated with earlier age at symptom onset, with clinical motor diagnosis being, on average, around the fourth decade.^3^ However, there are alterations in the brains of HD patients long before the emergence of symptoms, with imaging studies showing that striatal volumes differ between HD expansion carriers and controls since childhood, with initial striatal hypertrophy being followed by a rapid volume decline and atrophy.^4,5^ Recent evidence suggests that neurodevelopmental changes related to wild-type huntingtin (wtHTT) and mHTT, at the beginning of life, may also play a role.^6–10^

Cortical cell loss is a core feature of HD and numerous neuroimaging studies, across a range of modalities, have demonstrated cortical changes many years before disease onset in the premanifest stage.^11–16^ Moreover, analysis of post-mortem HD brains has shown that there is transcriptional dysregulation and somatic instability of the *HTT* CAG repeat in cortical neurons from HD expansion carriers.^17^ However, a recent study including children and adolescents using a region of interest (ROI) approach, did not find significant differences in cortical thickness between HD expansion carriers and controls,^18^ perhaps suggesting that these develop during early adulthood.^19^

The pathobiological processes underlying cortical cell loss are unclear. Unlike the end stages of HD, where post-mortem data may be used to explore disease mechanisms,^20^ premanifest HD (preHD) is more challenging due to a lack of post-mortem brains. Moreover, in animal models, CAG repeat lengths are typically much longer than in adult-onset HD and extrapolating conclusions from animal studies to non-juvenile HD patients is challenging.^21^

The advent of the Allen Human Brain Atlas (AHBA) has made this problem tractable. The AHBA provides brain-wide gene expression data for more than 20 000 genes using bulk tissue microarray analyses of 3702 distinct samples, being particularly focused on cortical grey matter. Gene expression data from the AHBA were mapped into the stereotaxic space,^22^ making it possible to link the spatial patterns of gene expression with anatomical variations in imaging metrics.^23^ Therefore the AHBA can be used to link cortical genetic topography to the cortical cell loss observed in preHD and inform the pathological processes that may be driving it.

We have previously used data from the AHBA to demonstrate that neurodegenerative white matter loss in preHD, 15 years from onset, is related to synaptic and metabolic genes.^24^ More recently we observed that increases in resting state functional MRI (fMRI) activity, associated with CSF neurofilament light, are specific to neuronal genes in HD gene carriers 25 years before onset.^25^

**Figure 1:**
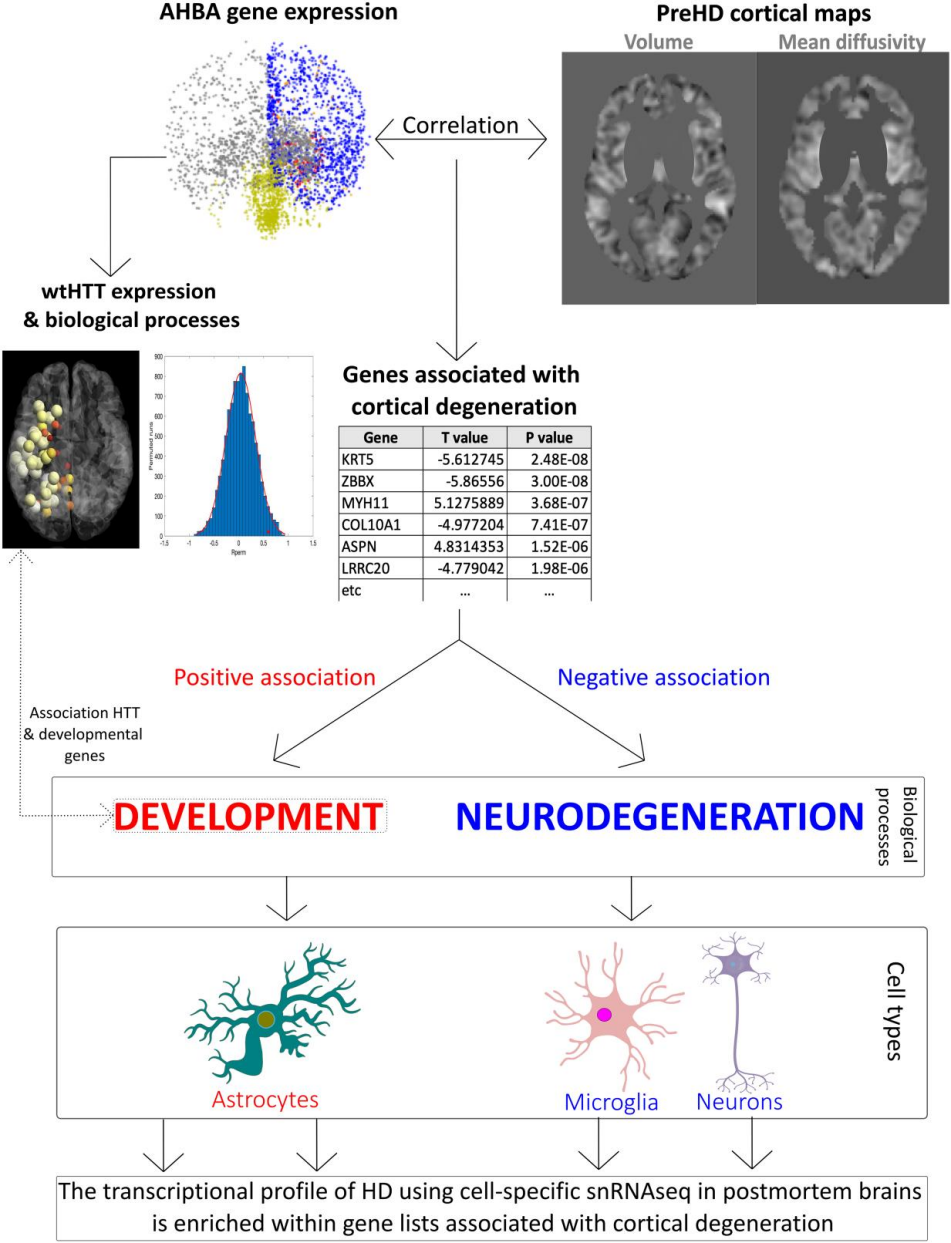
**Summary of analysis pipeline and main results.** T-maps examining areas with lower cortical volume and higher mean diffusivity (cortical cell loss) in premanifest Huntington’s disease (preHD) compared to healthy controls were obtained and correlated with Allen Human Brain Atlas (AHBA) data at the voxel-level. Gene lists positively and negatively associated with cortical cell loss in preHD were provided. Genes positively associated with cortical cell loss were involved in development and enriched in transcriptomic profiles from astrocytes. The expression of *HTT* in the healthy human brain was associated with developmental biological processes and correlated with the expression of developmental genes. Genes negatively associated with cortical cell loss were involved in neurodegenerative (synaptic and metabolic) biological processes, being enriched in transcriptional profiles from neurons and microglial cells. The cell-specific transcriptional profiles from post-mortem Huntington’s disease (HD) brains using single-nucleus RNA sequencing (snRNAseq) were enriched within gene lists associated with cortical cell loss, affecting the same cell types and in the same directions as the ones found in the EWCE analyses. wt = wild-type.

Here, we focus specifically on cortical grey matter cell loss in preHD, measured using volumetric and diffusion MRI. Previous HD studies, using data from the AHBA, have performed ROI analyses with lower spatial definition and depending on *a priori* selection of ROIs. Here, we employed a voxel-wise approach,^26^ which enables investigation at a much higher resolution than our previous work.^24,25^ We combined all genetic probes from the AHBA and voxel-wise imaging data to investigate the biological processes associated with grey matter cell loss in preHD (Fig. 1).

We show that both developmental and neurodegenerative processes are associated with cortical cell loss. Given the role of wtHTT in neurodevelopment, we test the hypothesis that the cortical expression of *HTT* and neurodevelopment genes are topographically related.

Finally, we also investigate the cell specificity of the developmental and neurodegenerative processes through expression-weighted cell-type enrichment (EWCE) using single nucleus RNAseq (snRNAseq) from HD post-mortem brains and healthy control subjects.

## Materials and methods

### Participants and data

A total of 239 participants were recruited for TrackOn-HD from four study sites (Leiden, London, Paris and Vancouver): 22 early HD, 106 preHD and 111 healthy control subjects. We used exclusively data from the baseline visit. Gene expansion carriers were required to have ≥40 CAG repeats in the *HTT* gene and a disease burden score (DBS) >250.^27^ Control participants were gene-negative volunteers and family members. At each visit, participants underwent a neuropsychological battery, clinical examination and a brain MRI. Inclusion criteria required age between 18 and 65 years free from major psychiatric, medical or neurological disorders and able to tolerate MRI. The study was approved by the local ethics committee and all participants provided written informed consent. For further details, see Kloppel *et al.*^28^

Early HD participants were removed from our analyses, as this study was focused on preHD only. Forty controls and 26 preHD participants were excluded because of incomplete clinical data, absent structural/diffusion imaging, or both. Excluded preHD participants were younger (included preHD, 43.88 ± 8.87, excluded preHD, 39.20 ± 7.95; *P* = 0.0184) but there were no differences in CAG repeat length (included preHD, 42.78 ± 2.24, excluded preHD, 43.58 ± 2.30; *P* = 0.1190) or disease burden score (included preHD, 303.22 ± 52.89, excluded preHD, 304.01 ± 51.85; *P* = 0.9471). There were no significant differences in age between included and excluded control subjects (included controls, 49.59 ± 9.69, excluded controls, 45.59 ± 11.82; *P* = 0.0566). There were no significant differences in the proportion of male and female subjects between included and excluded participants among controls or preHD participants.

The same participants were included in the volumetric and diffusion analyses.

### MRI data acquisition

Data were acquired on two different 3 T MRI scanner systems (Philips Achieva at Leiden and Vancouver and Siemens Trio at London and Paris). Scanning protocols were standardized between sites and inter-scanner comparisons were performed using human volunteers or phantoms. T_1_-weighted images were acquired using a 3D MP-RAGE acquisition sequence. T_1_ imaging parameters were (Siemens/Philips): repetition time = 2200/7.7 ms, echo time = 2.2/3.5 ms, flip angle = 10/8^°^, field of view = 28/24 cm, matrix size = 256 × 256/224 × 224, yielding 208/164 sagittal slices covering the entire brain with a slice thickness of 1.0 mm with no gap.

Diffusion-weighted images (DWI) were acquired with echo planar imaging sequences and 42 unique gradient directions (*b* = 1000 s/mm^2^). Seven images with no diffusion weighting (*b* = 0 s/mm^2^) or one image with no diffusion weighting were collected from the Siemens and Philips scanners, respectively. Diffusion imaging parameters were (Siemens/Philips): repetition time = 1300/1100 ms, echo time = 88/56 ms, voxel size = 2 × 2 × 2/1.96 × 1.96 × 2 mm^3^. Seventy-five slices were collected for each diffusion-weighted volume. For further imaging acquisition information, see Klöppel *et al*.^29^

### MRI data processing

#### Volumetric imaging

Baseline 3D T_1_ volumetric images were bias-corrected using the N3 algorithm^30^ and processed with SPM12 (https://www.fil.ion.ucl.ac.uk/spm/software/spm12/) running in MATLAB version R2012B. T_1_ images were segmented, and warped using DARTEL, incorporating a modulation step and smoothed at 4 mm full-width at half-maximum for the cross-sectional VBM analysis. All images were visually checked for processing artefacts after each step.

#### Diffusion-weighted imaging

DWI images were first brain extracted with FSL *bet* and corrected for eddy-current induced distortions and motion using FSL *eddy* (www.fmrib.ox.ac.uk/fsl).^31^ FSL *dtifit* was used to fit the diffusion tensor and derive mean diffusivity. Mean diffusivity maps, which describe the average diffusivity across all directions in mm^2^/s, were calculated from the diffusion tensor as the average of the three eigenvalues. Mean diffusivity reflects the freedom of water movement and is inversely related to cell density. Tensor metrics and orientations in all images were visually checked after each step to ensure accurate fitting.

NiftyReg^32^ was used to transform each subjects’ mean diffusivity map into standard Montreal Neurological Institute 152 (MNI152) space. First, the T_1_ image was brain extracted with FSL *bet*. Next, *reg_aladin* was used to align the T_1_ to the MNI template using an affine transformation. *reg_f3d* was then used to correct for residual inter-subject anatomical variation using a non-linear transformation. The mean diffusivity maps were then first linearly and then non-linearly registered to their T_1_ maps. Finally, the transformations were combined and applied to obtain the mean diffusivity maps into MNI space. The code is available in https://github.com/cestevezfraga. All images were visually checked to ensure accurate registration between mean diffusivity maps and MNI152.

We performed a Pearson's correlation between mean diffusivity and volume T values. The results were plotted using *ggplot* in Rstudio.

### Group differences in volume and diffusivity between premanifest Huntington’s disease patients and controls

Statistical parametric mapping (SPM) linear regression models were used to perform between group analyses (preHD versus controls) in SPM 12 for both grey matter volume and mean diffusivity. For each analysis this results in a test statistic (t-stat) for every voxel in the brain and can be visualized by SPM T-map, representing voxel t-stats in the brain. Typically, these maps are thresholded to correct for multiple comparisons when performing, for example, voxel-based morphometry. The objective of our analyses was to understand the relationship of group-level differences in volume/diffusion measures and how these relate to gene expression throughout the cortex, with the aim of understanding the biological processes associated with these changes. Therefore, the t-stats of all voxels in the cortex are used as opposed to just those that survive a statistical threshold. A similar voxel-wise approach was previously used by Altmann *et al*.^26^ and analogous ROI approaches have also been used with AHBA data.^33–35^

Age, sex and site were included as covariates in both analyses, while total intracranial volume was only included as a covariate in the volumetric analysis, as standard for diffusion and volumetric studies, respectively.^36,37^ Since HD patients have cortical volume loss,^38^ the unthresholded T-map evaluating areas where volume was lower in preHD than in controls was used for the analysis. In contrast, mean diffusivity is higher in HD compared to controls, indicating decreased structural organization.^39^ Therefore, the T-maps evaluating areas where mean diffusivity was higher in preHD than controls were used for the analysis.

**Figure 2:**
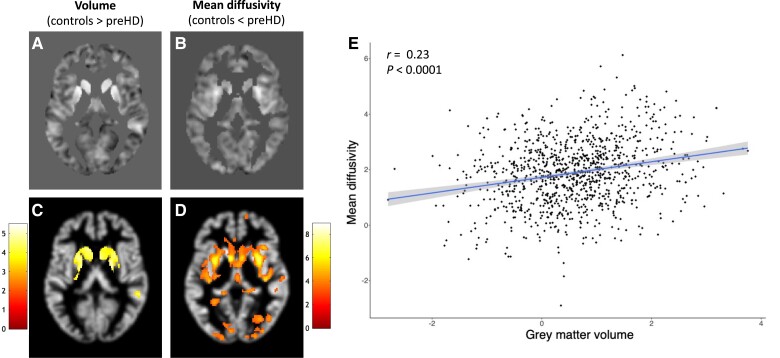
**Results of the imaging analyses comparing preHD and healthy controls.** T-maps showing the volumetric (**A**) and mean diffusivity (**B**) differences between premanifest Huntington’s disease (preHD) and controls, used in the transcriptomic analyses. Statistically significant differences in volume (**C**) and mean diffusivity (**D**) between preHD and controls, thresholded at *P* < 0.05 (family-wise error cluster-corrected, cluster forming threshold *P* = 0.001), overlaid onto the grey matter map of a representative participant. The colour bar represents T-scores. In **E** the associations between T-values for mean diffusivity and for cortical volume from **A** and **B** are represented in the voxels selected for the transcriptomic analysis.

Although the unthresholded T-maps were used for the association analysis, the T-maps were thresholded at *P* < 0.05 (family-wise error cluster-corrected, cluster forming threshold *P* = 0.001) to illustrate areas with significant differences in the directions tested between preHD and controls (i.e. lower volume and higher mean diffusivity in controls) (Fig. 2C and D).

### Association analysis

Gene expression data were extracted from the AHBA.^22^ The AHBA provides microarray expression data from six post-mortem brains (one female, age distribution 42.5 ± 13.38 years). Each sample has a total of 58 692 gene probes with associated coordinates in MNI152 space. The analysis was restricted to the left hemisphere since only two donors have available data from the right hemisphere, in line with previous analyses.^40^ Probes sampling more than one gene (*n* = 1512), mapped to DNA sequences between genes (*n* = 5013), not mapped to a region (*n* = 1569) or expressed in <300 cortical samples (*n* = 13 941) were excluded, leaving a total of 36 657 microarray probes covering 16 772 genes.

The correlation between scores from the cortical volume and mean diffusivity T-maps and gene expression from the AHBA were examined through Spearman correlations separately for each of the AHBA donors. The spatial eigenvector method was used to account for spatial autocorrelation.^26^

The T-scores and the *P*-value for the association between volume loss/increased diffusivity and gene expression between the different donors were combined into a single *P*-value using the weighted sums of the Z-scores. We centred our analysis on cortical regions from the AHBA.^40^

The code for regional association analysis and adjusting for spatial autocorrelation can be found at https://github.com/cestevezfraga. When one gene was measured in different probes, the average T-value was estimated. The resulting list was filtered to include only genes present in the AHBA database obtaining a total of 13 170 genes in the final dataset.

The individual genes associated with cortical volume loss and cortical mean diffusivity were summarized using a modified version of the volcano plots in Rstudio version 1.3.1093. Genes where different probes measuring expression from the same gene were associated with cortical features in opposite directions were removed from graphic representation in the volcano plots to facilitate visualization, but were included in the statistical analysis.

Positively correlated genes were those with positive T-values (i.e. higher expression associated with greater volume loss or higher mean diffusivity), while negatively correlated genes were these with negative T-values (i.e. lower expression associated with greater volume loss or higher mean diffusivity).

### Gene ontology analysis

To investigate the biological processes involved in cortical volume loss and mean diffusivity increases in preHD we performed Gene Ontology (GO) enrichment analyses in June 2022 to determine the biological processes significantly enriched in the top 10% of genes positively and negatively correlated with cortical volume loss and cortical mean diffusivity. The analyses were repeated including the top 20% and 30% of genes for consistency. GO analyses were adjusted for multiple comparisons using the Benjamini-Hochberg correction implemented in g:Profiler with a significance threshold of 0.05. An in-house dictionary was used to manually summarize the significant GO terms into broader categories to facilitate the interpretation of the results. The complete list of GO terms obtained from enrichment and classified into categories is available in the Supplementary material.

### Topographic association between the expression of wild-type HTT and developmental genes

We investigated the relationship between the expression of wt*HTT* and developmental genes in the healthy brain by first obtaining a list of genes involved in human neurodevelopment from Kang *et al*.^41^ Here, the exon-level transcriptomes from 58 post-mortem human brains free from neurological disease were analysed using an Illumina 2.5 million single nucleotide chip in 16 brain regions, including 11 neocortical areas. Brain development was divided into 15 periods encompassing from embryonic (<4 weeks postconceptional to <8 weeks postconceptional) to late adulthood (>60 years) stages.

We included the 10 genes (‘developmental genes’) differentially expressed in the neocortex between early mid-fetal (from 13 postconceptional weeks) and late infancy (until 6 months of age) as these stages are critical for cortical development. Next, we obtained the expression from *HTT* and the expression from the 10 developmental genes from a 110 ROI atlas, combining Desikan^42^ cortical and subcortical regions with the Diedrichsen cerebellar atlas^43^ using the abagen toolbox^44^ (github.com/rmarkello/abagen). To obtain gene expression, the tissue samples were matched to one of the 110 ROIs using AHBA MRI data. Probes were filtered based on their expression relative to background noise. Probes with expression levels above the background in over 50% of samples were chosen. To represent each gene, the probe with the highest intensity value was selected. Data between homologous cortical areas were combined. Samples were assigned to brain regions using a 2 mm threshold from a given parcel. After normalizing the gene expression data across genes using the robust sigmoid function, a total of 15 633 genes were included in the dataset. Samples assigned to the same brain region were averaged separately for each donor and then across donors. The expression of the 10 developmental genes were extracted from this dataset. This resulted in a 10 × 110 gene-ROI matrix.

Principal component analysis was then performed on this matrix. This principal component analysis provided a value for the first principal component, representing the expression of developmental genes in each ROI. The first component was then correlated with the expression of *HTT* in the same ROIs. Next, we investigated the relationship between the expression of *HTT* and the expression of developmental genes through spatial permutation testing. We generated permutation maps from the cortical ROIs based on sphere rotations.^45^ This process was repeated for 10 000 random permutations developing a spatially correlated null distribution. Next, the Spearman correlations between *HTT* expression and the expression of the first component of the developmental genes were estimated in the spatially permutated maps. *P*-spin values were calculated based on the explained variance in the observed data relative to the variance explained in the null model. The statistical significance was tested with two-tailed *α* = 0.05. The code used for the spatial permutation analysis is available in https://github.com/cestevezfraga.

We also examined the significance of the association between *HTT* expression and the expression of the first component of the 10 developmental genes using a complementary approach through bootstrapping. We generated 10 000 permutations of random gene lists of equal size to the developmental gene list from the AHBA and obtained their expression from the same 110 ROIs. Principal component analysis was performed on the random gene lists and the first component was correlated with *HTT* expression. This bootstrapping approach was used to obtain a distribution of correlation coefficients from the random gene lists against which the correlation between *HTT* and the expression of developmental genes was compared for statistical inference. The statistical significance was tested with two-tailed *α* = 0.05.

Finally, we used the CoExp platform (https://rytenlab.com/coexp) to perform co-expression network analysis^46^ investigating the modules of genes associated with *HTT*. This software can be used to examine specific areas known to be affected by HD. Co-expression networks were therefore investigated in the two cortical regions (frontal and occipital cortex) previously shown to be involved in HD,^15,38,47–49^ with samples available from the UK Brain Expression Consortium (UKBEC) (https://ukbec.wordpress.com/), including 134 human brains of European descent free of neurodegenerative disorders generated using Affymetrix Exon 1.0 ST Arrays.^50^

### Expression weighted cell-type enrichment analysis

To investigate the cell types associated with cortical volume loss and mean diffusivity increases we used EWCE.^51^ The top 10% of genes positively and negatively associated with the T-maps were used as target lists. The analyses were repeated including the top 20% and 30% of genes for consistency, available in the Supplementary material.

*P*-values are based on the 100 000 permutations controlling for transcript length and guanine-cytosine content. Results were corrected for multiple comparisons using the Benjamini-Hochberg method, as standard for EWCE. Single cell RNAseq data from the middle temporal gyrus were used from the AHBA.^52^ The analyses were replicated using the same parameters in a different dataset, where cell-specific transcriptomic profiles were obtained using DroNC seq from human samples.^53^ The EWCE package can be accessed in https://github.com/NathanSkene/EWCE.

### Enrichment analyses of cell-specific genes showing differential expression in Huntington’s disease

We used data from a snRNAseq study to examine differential gene expression across various brain cell types in individuals with HD compared to control subjects. The dataset specifically focused on the anterior cingulate cortex and was obtained from Al-Dalahmah *et al*.^54^ Al-Dalahmah *et al*.^54^ examined cell-specific gene expression in six HD brains (three females, CAG: 46.83 ± 2.23, age: 52 ± 11.33) and six controls (two females, age: 52.67 ± 8.84) identifying the transcriptional signatures for neurons, astrocytes, endothelial cells, oligodendrocytes and oligodendrocyte precursors.

We investigated whether the cell-specific transcriptomic pattern for each cell type (neurons, astrocytes, endothelial cells, oligodendrocytes and oligodendrocyte precursors) using snRNAseq in HD brains was enriched in the top 10% of genes positively and negatively correlated with cortical volume loss and cortical mean diffusivity increases. We used a hypergeometric distribution to assess the probability of the overlap in each cell type through the *phypher* command in Rstudio.

## Results

The demographic characteristics of study participants at baseline are shown in Table 1. As expected, controls were significantly older than preHD participants (controls 49.59 years, preHD: 43.88 years. *P* = 0.0002). However, the effect of HD and the effect of ageing are in the same direction (i.e. higher mean diffusivity and lower volume with ageing) and age was included as a covariate in all analyses.

**Table 1:** Demographic characteristics of study participants

	Controls	PreHD	*P*-value
*n*	71	80	N/A
Age	49.59 ± 9.69	43.88 ± 8.87	**0.0002**
Sex (M:F)	29:42	38:42	0.4113
Study site	Leiden: 22 London: 22 Paris: 11 Vancouver: 16	Leiden: 25 London: 21 Paris: 17 Vancouver: 17	N/A
CAG	–	42.78 ± 2.24	N/A
DBS	–	303.22 ± 52.89	N/A

Group comparisons were made using *t*-tests (age, DBS) and chi square tests (sex). Significant differences (*P* < 0.05) are in bold.CAG = cytosine-adenine-guanine; DBS = disease burden score; N/A = not applicable; preHD = premanifest Huntington’s disease.

### Imaging analysis

PreHD participants had large areas of volume loss compared to healthy controls, mainly affecting the striatum and diffuse cortical areas bilaterally. These results survived cluster correction at a *P* = 0.05 (cluster-forming threshold *P* = 0.001). The unthresholded SPM T-map showing larger volume loss in preHD compared to controls was used for the transcriptomic analyses (Fig. 2).

PreHD participants had large areas of increased mean diffusivity compared to controls, particularly in the striatum and occipital cortex. These results survived cluster correction at *P* = 0.05 (cluster forming threshold *P* = 0.001). The SPM T-map showing increased mean diffusivity in preHD compared to controls was used for the transcriptomic analysis (Fig. 2).

There was a positive correlation (*r* = 0.23, *P* < 0.0001) between the T-values for mean diffusivity increases and cortical volume loss in preHD, indicating that higher mean diffusivity is associated with lower volume (Fig. 2).

### Gene expression associated with cortical cell loss

**Figure 3:**
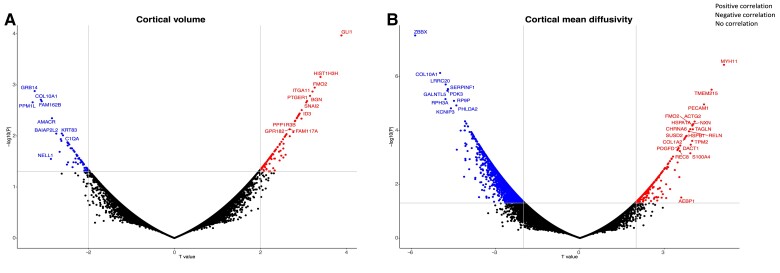
**Scatterplot representing the genes associated with cortical cell loss.** The y axis represents the logarithmic of the P value while the x axis shows the T value. The horizontal line represents the threshold for significance while the vertical lines show the T = 2 threshold for gene expression positively (right side) or negatively (left side) correlated with cortical volume loss (**A**) and increases in cortical diffusivity (**B**). The “*ggrepel*” package in Rstudio was used to avoid label overlap.

Gene-MRI association analysis revealed 686 genes positively and 632 gene negatively associated with cortical volume loss in preHD. In contrast, gene-MRI association analysis showed 347 genes positively and 971 genes negatively associated cortical mean diffusivity (Supplementary material). The results are represented in Fig. 3.

### Gene ontology enrichment analyses

#### Genes involved in development are positively correlated with cortical cell loss in premanifest Huntington’s disease

**Figure 4:**
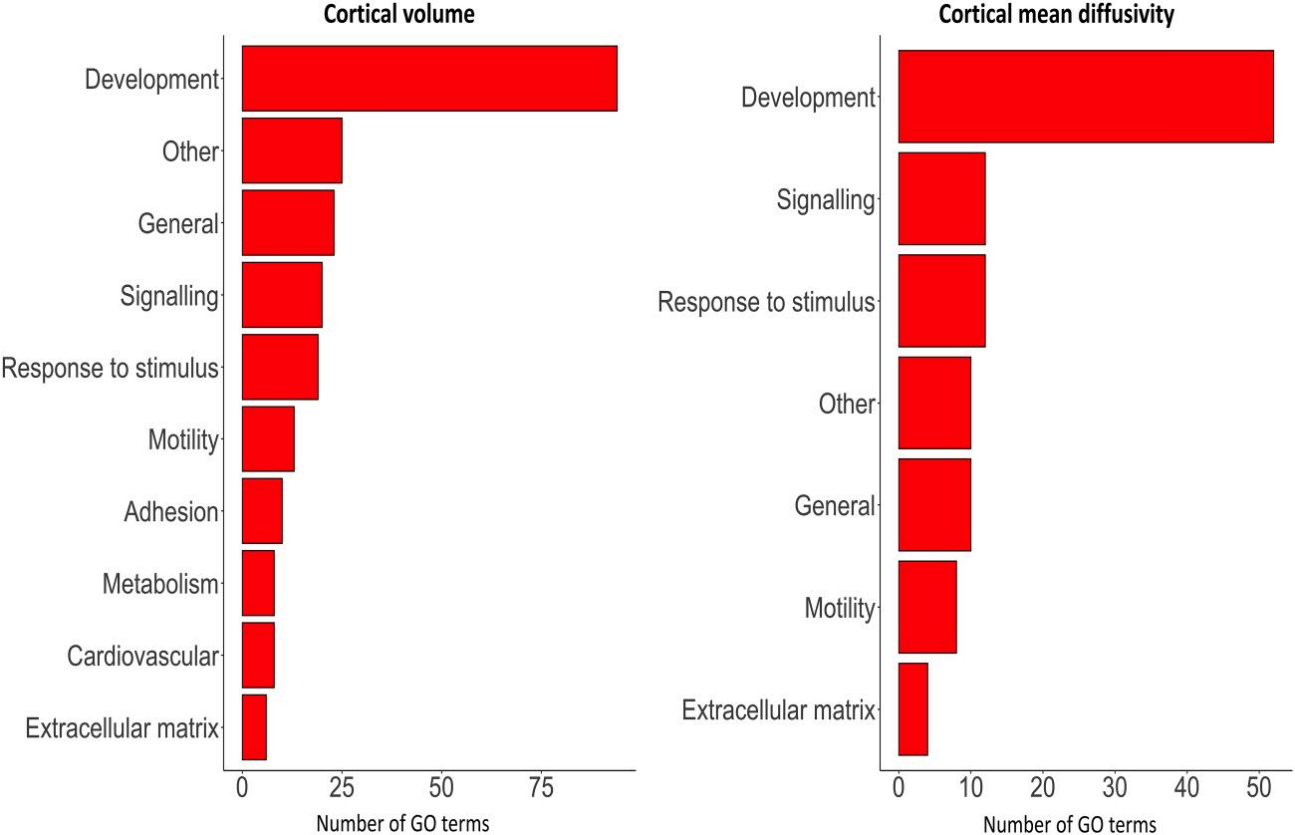
**Total number of GO biological process terms positively associated with cortical cell loss in premanifest Huntington’s disease.** Each row represents the number of GO terms involved within the module. GO = gene ontology.

Development encompassed the largest number of GO terms for genes positively correlated with greater atrophy and increased diffusivity (developmental profile), using the in-house dictionary, with the largest proportion of significant GO terms for cortical volume: 41.96%; and for cortical mean diffusivity: 46.02% (Fig. 4). Signalling (cortical volume: 8.93% cortical mean diffusivity: 10.53%), response to stimulus (cortical volume: 8.48%, cortical mean diffusivity: 10.53%) and motility (cortical volume: 5.80%, cortical mean diffusivity: 7.08%) were also present in both analyses.

Two hundred and twenty-four GO terms were significantly enriched in genes positively correlated with cortical volume and 113 with cortical mean diffusivity. The top five GO terms positively associated with cortical volume loss were ‘multicellular organismal process’ (*P* = 1.21 × 10^−27^), ‘animal organ development’ (*P* = 2.17 × 10^−27^), ‘anatomical structure development’ (*P* = 2.07 × 10^−26^), ‘multicellular organism development’ (*P* = 5.51 × 10^−26^) and ‘anatomical structure morphogenesis’ (*P* = 2.83 × 10^−25^). The top five positive GO terms positively correlated with cortical mean diffusivity were ‘anatomical structure development’ (*P* = 1.54 × 10^−15^), ‘developmental process’ (*P* = 2.80 × 10^−15^), ‘tissue development’ (*P* = 1.91 × 10^−14^), ‘multicellular organism development’ (*P* = 1.96 × 10^−15^) and ‘system development’ (*P* = 9.88 × 10^−14^). Eight out of the 10 top GO terms positively correlated with cortical cell loss were shared between the cortical mean diffusivity and cortical volume analyses. The results were consistent when analysing the top 10%, 20% and 30% of genes positively correlated with cortical cell loss (Supplementary material).

#### Metabolic and synaptic genes are negatively associated with cortical cell loss

**Figure 5:**
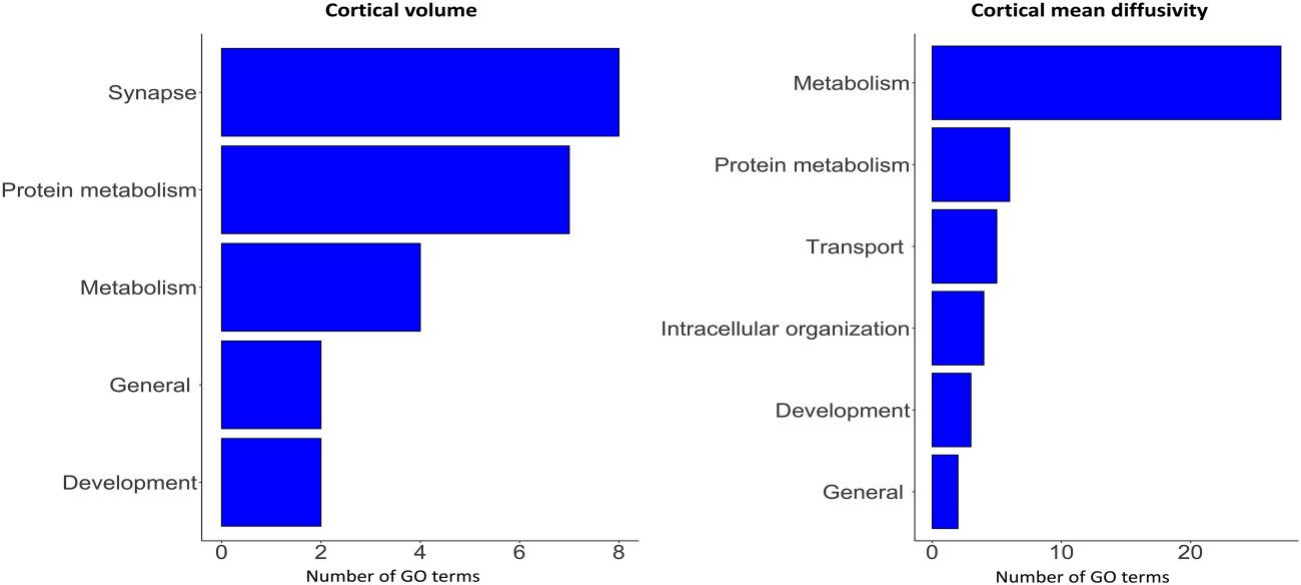
**Total number of GO biological process terms negatively associated with cortical cell loss in premanifest Huntington’s disease**. Each row represents the number of GO terms involved within the module. GO = gene ontology.

Metabolism and synaptic function categories encompassed the largest number of GO terms for genes negatively correlated with greater atrophy and increased diffusivity (neurodegenerative profile), using the in-house dictionary (Fig. 5), with GO terms for metabolism (proportion of significant GO terms; cortical volume: 18.18%, cortical mean diffusivity; 57.45%) and protein metabolism (cortical volume: 31.81%, cortical mean diffusivity: 12.76%) being present in both the cortical volume and cortical mean diffusivity analyses. GO terms involved in synaptic function were only negatively associated with cortical volume (36.36%).

Twenty-two GO terms were significantly enriched in genes negatively correlated with cortical volume and 47 with cortical mean diffusivity. The top five GO terms enriched in genes negatively correlated with cortical volume were ‘cellular macromolecule metabolic process’ (*P* = 2.06 × 10^−6^), ‘organonitrogen compound metabolic process’ (*P* = 1.38 × 10^−5^), ‘protein metabolic process’ (*P* = 8.52 × 10^−5^), ‘cellular protein metabolic process’ (*P* = 9.96 × 10^−5^) and ‘phosphorus metabolic process’ (*P* = 3.73 × 10^−4^). The top five significant GO terms associated with cortical mean diffusivity were ‘cellular respiration’ (*P* = 1.26 × 10^−14^), ‘mitochondrion organization’ (*P* = 2.54 × 10^−14^), ‘mitochondrial ATP synthesis coupled electron transport’ (*P* = 1.93 × 10^−13^), ‘ATP synthesis coupled electron transport’ (*P* = 1.93 × 10^−13^) and ‘energy derivation by oxidation of organic compounds’ (*P* = 4.21 × 10^−14^). The results were consistent when analysing the top 10%, 20% and 30% of genes negatively correlated with cortical cell loss (Supplementary material).

### Association between HTT expression and the expression of developmental genes

**Figure 6:**
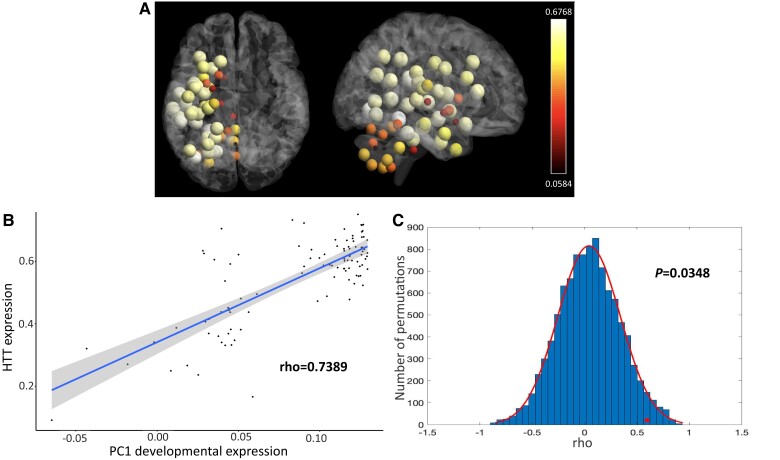
**Association between *HTT* expression and developmental genes.** Average expression of the *HTT* in the Allen Human Brain Atlas (AHBA), left hemisphere only (*n* = 6). Expression normalized with the Abagen toolbox and depicted in the Desikan (cortical and subcortical) and Diedrichesen (cerebellar) atlases using BrainNet Viewer (**A**). The genes involved in early brain development obtained from Kang *et al*.^41^ and also present in the AHBA dataset were: *CYP26A1*, *FBXW7*, *MCHR2*, *OSTN*, *PART1*, *RORB*, *SATB2*, *SSX2IP*, *TMS6SF1* and *TSHZ3*. The correlation between *HTT* expression from the AHBA and the first component of the expression of developmental genes is shown in **B**. In **C**, the distribution of the correlations between *HTT* expression and the first principal components component of the expression of random sets of 10 genes from the AHBA through bootstrapping (resampling 10 000 times) is represented. The red circle illustrates the association between *HTT* expression and correlation with the first principal components analysis component of developmental genes from Kang *et al*.^41^ The *y*-axis represents the number of permutations of random genes from the AHBA list. The *x*-axis represents the correlation coefficients.

Following our results showing a consistent positive association between cortical cell loss and genes enriched in developmental GO terms (Fig. 6), we next investigated the relationship between the expression of wt*HTT* and developmental processes.

Genes differentially expressed in the human cortex during the developmental period were obtained from Kang *et al*.^41^ We performed principal component analysis on expression of developmental genes from the AHBA. The first component explained 35.12% of the variance (Supplementary material) and was significantly correlated with the expression of *HTT* in the AHBA through spatial permutation testing (rho = 0.5412, Pspin < 0.0001). The correlation was present also following resampling 10 000 times using the bootstrap method (rho = 0.5956, *P* = 0.0348) (Fig. 6).

Next, transcriptomic data from post-mortem healthy brains were used to investigate the biological processes associated with genes co-expressed with *HTT* with CoExp in the occipital and frontal cortex. *HTT* expression was significantly associated with the expression of genes enriched in GO terms involved in development in the occipital cortex (‘regulation of neuron projection development’, *P* = 5.26 × 10^−5^; ‘nervous system development’, *P* = 5.96 × 10^−5^; ‘regulation of neuron differentiation’, *P* = 7.82 × 10^−5^) as well as ion transport (‘ion transport’, *P* = 0.000298) and synapse (‘modulation of chemical synaptic transmission’, *P* = 5.19 × 10^−5^).

In the frontal cortex, *HTT* expression was significantly associated with the expression of genes enriched in GO terms involved in protein metabolism (‘protein modification process’, *P* = 0.0299, ‘cellular protein modification process’, *P* = 0.0299) but not with developmental genes.

### Expression-weighted cell type enrichment analysis

**Figure 7:**
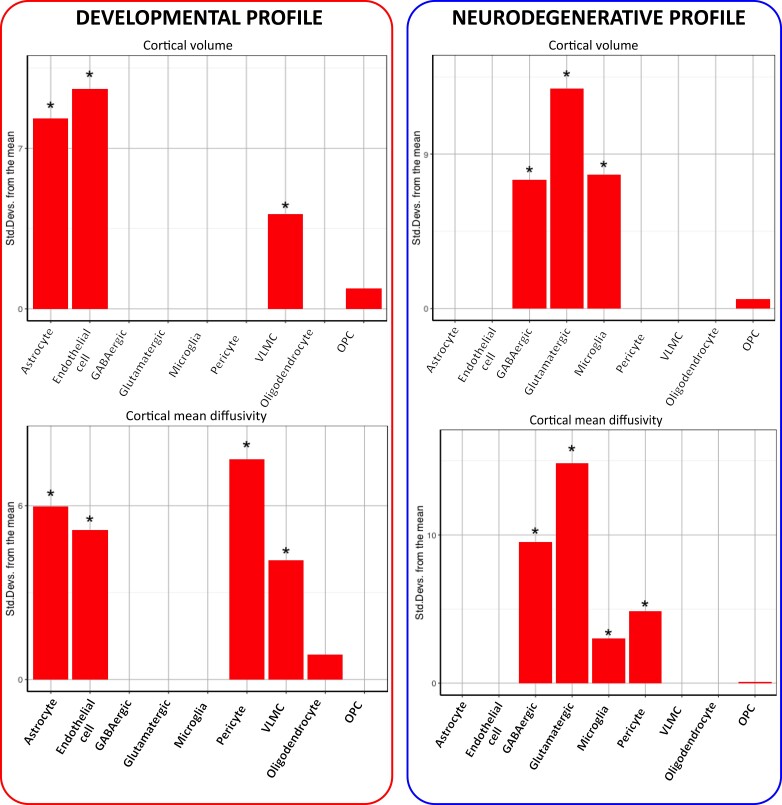
**EWCE analysis examining cell types enriched within the developmental and neurodegenerative profiles using the AHBA single-cell transcription dataset**. Data are presented as standard deviations from the mean. *Statistically significant. AHBA = Allen Human Brain Atlas; EWCE = expression weighted cell type enrichment; OPC = oligodendrocyte precursor cells; VLMC = vascular leptomeningeal cell.

Next, we performed EWCE in the 10% of genes positively (developmental profile) and 10% of genes negatively (neurodegenerative profile) associated with cortical cell loss (Fig. 7).

Genes positively correlated with cortical cell loss (developmental profile) were significantly associated with astrocytes (cortical volume, *P* < 1 × 10^−10^, cortical mean diffusivity, *P* < 1 × 10^−10^), endothelial cells (cortical volume, *P* < 1 × 10^−10^, cortical mean diffusivity, *P* < 1 × 10^−10^) and vascular leptomeningeal cells (cortical volume, *P* = 4 × 10^−5^, cortical mean diffusivity, *P* = 1 × 10^−4^), while pericytes were only enriched in the cortical mean diffusivity gene list *P* < 1 × 10^−10^).

Genes negatively correlated with cortical cell loss (neurodegenerative profile) were significantly enriched in GABAergic (cortical volume, *P* < 1 × 10^−10^, cortical mean diffusivity, *P* < 1 × 10^−10^) and glutamatergic neurons (cortical volume, *P* < 1 × 10^−10^, cortical mean diffusivity, *P* < 1 × 10^−10^) and in microglial cells (cortical volume, *P* < 1 × 10^−10^, cortical mean diffusivity, *P* = 0.00189), while pericytes were only enriched in the cortical mean diffusivity analysis (*P* < 1 × 10^−10^).

The results were consistent when analysing the top 10%, 20% and 30% of genes positively and negatively correlated with cortical cell loss. Similarly, we replicated the results using the DroNC database (Supplementary material).^53^

### Enrichment analyses of cell-specific genes showing differential expression in Huntington’s disease

In line with the EWCE analysis, genes with differential expression in astrocytes using snRNAseq from HD post-mortem brains were significantly enriched among genes positively (developmental profile) correlated with cortical volume (*P* = 0.0164) and cortical mean diffusivity (*P* = 5 × 10^−4^).

Also consistent with the EWCE analysis, genes with differential expression in HD neurons were significantly enriched among genes negatively (neurodegenerative profile) correlated with cortical volume (*P* = 0.0035) and cortical mean diffusivity (*P* = 7.33 × 10^−5^). Similarly, genes with differential expression in microglia from HD brains were significantly enriched among genes negatively correlated with cortical volume (*P* = 0.0014) and with cortical mean diffusivity (*P* = 3.34 × 10^−4^).

In summary, the developmental gene profile shows astrocyte cell type-specific relationships in both EWCE and snRNAseq data, while the neurodegenerative profile shows neuronal and microglia cell-specific relationships in both EWCE and snRNAseq data. This suggests the involvement of distinct cell types in the developmental and neurodegenerative components of HD.

## Discussion

We investigated how cortical genetic topography relates to cortical cell loss in a large sample of preHD participants using a multi-modality high resolution voxel-based imaging approach *in vivo* and examined the cell-specific relationships using snRNAseq data from post-mortem HD and control brains. Cortical cell loss was positively correlated with the expression of developmental genes and negatively correlated with the expression of synaptic and metabolic genes, which have been implicated in neurodegeneration. Given the role of wtHTT in neurodevelopment^55^ we demonstrate a topographical relationship between wt*HTT* and developmental genes. We also identified distinct cell-specific relationships where genes in the development profile were enriched for astrocyte-specific genes differentially expressed in HD, whereas genes in the neurodegenerative profile were enriched for neuronal and microglial-specific genes differentially expressed in HD.

We show that areas with higher expression of developmental genes are associated with greater cortical cell loss in HD. These findings were consistent across both imaging modalities, with 40% of significant GO terms being involved in development, pointing towards increased vulnerability of regions with prominent development roles. In addition, we showed that the expression of the wtHTT protein in the healthy human brain is significantly associated with the expression of genes involved in early stages of human neurodevelopment. Moreover, co-expression network analysis revealed that developmental genes are co-expressed with *HTT* in the occipital cortex, a region undergoing the greatest rate of cortical atrophy in HD during motor conversion.^15^ These findings suggest that lower wtHTT expression in HD expansion carriers during development, due to the presence of a single wild-type allele, may render specific areas vulnerable to neurodegeneration in adulthood.

The wtHTT protein has multiple functions and plays a crucial role during early brain development. The expression of wt*HTT* in the healthy human fetal striatum regulates the formation of the lateral ganglionic eminence, from which medium spiny neurons (MSNs) are generated.^56^ Consistently, homozygous deletions in the mouse homologue of the *HTT* gene result in embryonic lethality.^57^ Decreasing HTT expression from the late embryonic or early postnatal period in mice causes severe cortical and striatal malformations and perinatal lethality,^58^ while mice where the expression of wtHTT is severely reduced (∼15% of normal levels) exclusively during the developmental period, being restored after postnatal Day 21, develop abnormal striatal compartmentalization into striosomes and matrix during development, followed by neurodegeneration in the cortex and striatum during adulthood.^59^ Moreover, rare individuals with compound heterozygous loss-of-function variants in *HTT* present with a complex neurodevelopmental syndrome.^60^ These studies indicate that the wtHTT protein is critical during early neurodevelopment.

Most HD patients carry the *HTT* expansion in one allele, together with a single copy of the wild-type allele, resulting in reduced levels of wtHTT.^61,62^ This may explain the higher frequency of neurodevelopmental malformations compared to controls in post-mortem brains from HD patients.^63^ Similarly, although clinical manifestations of HD emerge on average, during mid-adulthood, there are numerous cellular abnormalities in the cortex of human HD fetuses, including changes in cell polarity and neuronal differentiation,^7^ and there is imaging evidence supporting the presence of structural brain changes in infants with the *HTT* mutation decades before expected clinical motor onset.^4,64^ Moreover, total intracranial volume (including tissue and CSF volume inside the calvarium), is also smaller in *HTT* gene expansion carriers compared with controls, not being associated with disease burden, and therefore suggesting abnormal development in the presence of the mutation.^65^ Single-nucleotide polymorphisms in *HTT* resulting in decreased transcription of the wild-type allele in HD patients, hasten age at onset.^66^ Also, a previous study investigating mRNA expression in the prefrontal cortex of HD brains showed that differentially expressed genes were involved in development both using functional clustering networks and GO enrichment analysis.^67^

Moreover, a recent study analysed the striatum of HD human and HD mouse brains finding that the striosomal compartment is more severely depleted of MSNs than the matrix compartment.^68^ Single nucleus RNAseq analysis of the same samples showed that the loss of the transcriptional differentiation of the striosome-matrix compartments is more prominent than the dysregulation of the transcriptomic distinction between indirect and direct MSNs, suggesting a hierarchy of vulnerability within striatal MSNs. Based on these results, the authors propose that striatal atrophy in HD might arise as a consequence of decreased wtHTT expression (together with the presence of mHTT) leading to abnormal compartmentalization in the striatum between striosomes and matrix during development. These changes would be compensated for until later stages when the differential depletion of indirect pathway MSNs in the striatum would lead to the emergence of motor symptoms. Interestingly, developmental and synaptic GO terms were also enriched in striosomal, matrix, D1 and D2 MSN genetic markers in the same study.

These results are consistent with our findings and suggest that the effects of decreased wtHTT alongside mHTT expression lead to cellular loss in the cortex and the striatum through similar mechanisms. Cell loss in HD could therefore be a consequence of abnormal development, which is compensated for until adulthood when developmentally vulnerable regions are overwhelmed by the presence of the mHTT protein and its downstream effects.^10,60^

The developmental transcriptomic profile in our analysis was enriched for astrocyte-specific genes differentially expressed in HD. Astrocytes are one of the most common cellular populations in the brain, being involved in neuronal homeostasis and blood–brain barrier (BBB) formation.^69^ There is increased expression of wt*HTT* during the embryonic and early postpartum period in HD mouse models, being particularly marked in astrocytes, with a 7-fold increase in wt*HTT* mRNA expression.^70^ Astrocytic dysfunction in HD mice results in reduced levels of the GLT-1 transporter during the first weeks of life,^71^ leading to increased glutamate in the synaptic space, causing secondary excitotoxicty.^72^ Similarly, abnormal excitatory activity has been found in HD mice during the early postnatal period. Normalizing abnormal excitatory transmission in HD mice during the early postnatal period with an ampakine, increasing the responsiveness of glutamate-binding AMPA receptors, restores neuronal synaptic function and prevents brain atrophy and abnormal phenotypes at later stages.^9^

The developmental profile was also enriched for genes specific for endothelial and vascular cells. Genes involved in influx and outflux mechanisms across the BBB are expressed at higher levels in the healthy developing brain and transport systems show increased function in the embryo compared with the adult,^73^ supporting the essential role of the BBB during early development. In HD, the transcriptomic signature of endothelial cells is altered,^74^ resulting in impaired tight junction formation and dysfunction of the BBB.^75^ In consequence, increased permeability of the BBB results in leakage of proinflammatory cytokines from circulation into the brain parenchyma, which could be responsible for increased inflammation in HD during mid-adulthood.^76,77^

With regards to the neurodegenerative profile, we show that regions with lower expression of genes involved in synaptic and metabolic function have greater grey matter cortical cell loss. This is consistent with our previous work showing that expression of synaptic and metabolic genes is associated with loss of white matter connections in preHD.^24^ Furthermore, transcriptomic studies in HD post-mortem brains have demonstrated that the main biological pathways associated with transcriptional dysregulation were involved in metabolic and synaptic function.^78,79^ Taken together, these findings suggest that mHTT-related transcriptomic dysregulation of synaptic and metabolic genes results in grey and white matter neurodegeneration.

The neurodegenerative profile was enriched for neuronal-specific genes differentially expressed in HD. Somatic instability, one of the core disease mechanisms in HD, is specific to neurons, particularly in the cortex and striatum.^80,81^ The neuronal damage induced through somatic instability is cumulative, leading to progressive increases in CAG repeat length and being associated with the onset of symptoms.^82^ These cumulative alterations, in combination with increased concentrations of mHTT during adulthood could lead to neuronal dysfunction with defects in ATP production and decreased transport of mitochondria to synapses,^83,84^ altered proteasomal degradation^85^ and inefficient autophagosome-lysosome fusion.^86^ Eventually these alterations would lead to the death of neuronal populations primed during development.

Microglial-specific genes differentially expressed in HD, were also enriched in the neurodegenerative profile. Microglial cells regulate synaptic pruning^87^ and abnormal synapse elimination by microglial cells is a mechanism present in multiple neurodegenerative conditions, including HD.^88^ There is excessive microglial reactivity and impaired migration of microglial cells^89^ underlying immune dysfunction in HD.^90^ The extensive evidence pointing towards neuroinflammation being a core mechanism for cell loss led to the development of the LEGATO HD and SIGNAL-HD clinical trials, administering drugs targeting inflammation to HD patients. These studies did not meet their clinical primary outcomes but showed significant improvements in caudate atrophy rates, perhaps through ameliorating mHTT-induced inflammation in the short term.^91,92^

We acknowledge the limitations of the AHBA atlas, in that it is derived from neurotypical individuals and not those with preHD. However, analyses of post-mortem data from the brains of HD patients and HD animal models have shown that transcriptional dysregulation is prominent in the striatum, with 515 genes differentially expressed. In contrast, only 25 genes show differential expression in the HD cortex.^20,93^ Therefore, we have restricted our analyses using exclusively cortical expression from the AHBA, where the transcriptional pattern of preHD is likely to be largely similar to that of healthy controls.^22^ Ideally, we would like to investigate genetic topography in preHD patients in post-mortem brains. However, average age at onset in HD is around 40 years of age with gene expansion carriers being typically healthy before symptoms emerge. Therefore, it is extremely challenging to obtain large datasets of post-mortem brains from preHD individuals.

There were significant differences in age between controls and preHD participants in our study. This is inherent to the age-dependent phenoconversion in HD. To account for this, age was added as a covariate in all imaging analyses. Also, a substantial proportion of controls were excluded from our analysis due to incomplete data collection. However, these subjects did not show significant differences compared to the included controls.

In summary, our work provides a link between developmental and neurodegenerative processes in HD. Based on these observations, we propose that HD could result as a two-step process targeting initially brain areas not developed normally due to loss-of-function of wtHTT and/or interference of mHTT in normal development, followed by neurodegeneration caused by gain-of-function of the mHTT protein. These alterations would result in neuronal loss that is initially compensated for through increased functional activity until these mechanisms fail, resulting in the emergence of clinical symptoms, as shown in our earlier work.^12^

## Supplementary Material

awad275_Supplementary_Data

## Data Availability

Data will be made available upon reasonable request. Code for analysis is publicly available at https://github.com/cestevezfraga.
