# Supplementary material for: Genetic topography and cortical cell loss in Huntington's disease link development and neurodegeneration
Source: Brain. 2023 Aug 17;146(11):4532–46. doi: 10.1093/brain/awad275 (PMC10629790; doi:10.1093/brain/awad275)
Supplement: awad275_Supplementary_Data [file awad275_supplementary_data.pdf]

Genetic topography and cortical cell loss in Huntington's disease link development and neurodegeneration

Supplementary Data

Figure 1 Map depicting the cortical T values obtained from the volumetric analysis comparing HD expansion carriers and controls. 2
Figure 2 Map depicting the cortical T values obtained from the mean diffusivity analysis comparing HD expansion carriers and controls. 3
Figure 3 Density plots illustrating the distribution of T values from the volumetric (left) and mean diffusivity (right) analyses comparing HD expansion carriers and controls 5
Figure 4 Summary of the analysis between HTT expression and developmental genes 6
Figure 5 Principal component analysis of developmental genes expression in the AHBA 84

Table 1 Top 10% genes positively associated with cortical volume 7
Table 2 Top 10% genes negatively associated with cortical volume 24
Table 3 Top 10% genes positively associated with cortical mean diffusivity 39
Table 4 Top 10% genes negatively associated with cortical mean diffusivity 48
Table 5 GO terms enriched in the top 10% of genes positively associated with cortical volume in preHD 71
Table 6 GO terms enriched in the top 10% of genes negatively associated with cortical volume in preHD 77
Table 7 GO terms enriched in the top 10% of genes positively associated with cortical mean diffusivity in preHD 78
Table 8 GO terms enriched in the top 10% of genes negatively associated with cortical mean diffusivity in preHD 81
Table 9 List of genes involved in neurodevelopment 83
Table 10 EWCE analysis showing cell types enriched within 10%, 20% and 30% genes positively associated with cortical volume loss using the AIBS single-cell transcription and the DroNC datasets 85
Table 11 EWCE analysis showing cell types enriched within 10%, 20% and 30% genes negatively associated with cortical volume loss using the AIBS single-cell transcription and the DroNC datasets 87
Table 12 EWCE analysis showing cell types enriched within the top 10%, 20% and 30% genes positively associated with increases in cortical mean diffusivity using the AIBS single-cell transcription and the DroNC-seq datasets 89
Table 13 EWCE analysis showing cell types enriched within the top 10%, 20% and 30% of genes negatively associated with increases in cortical mean diffusivity in the AIBS single-cell transcription and the DroNC-seq datasets 91

Figure 1 Map depicting the cortical T values obtained from the volumetric analysis comparing HD expansion carriers and controls.

The colorbar represents the magnitude of the T scores (white: black, higher: lower). The T values are unthresholded. HD, Huntington's disease

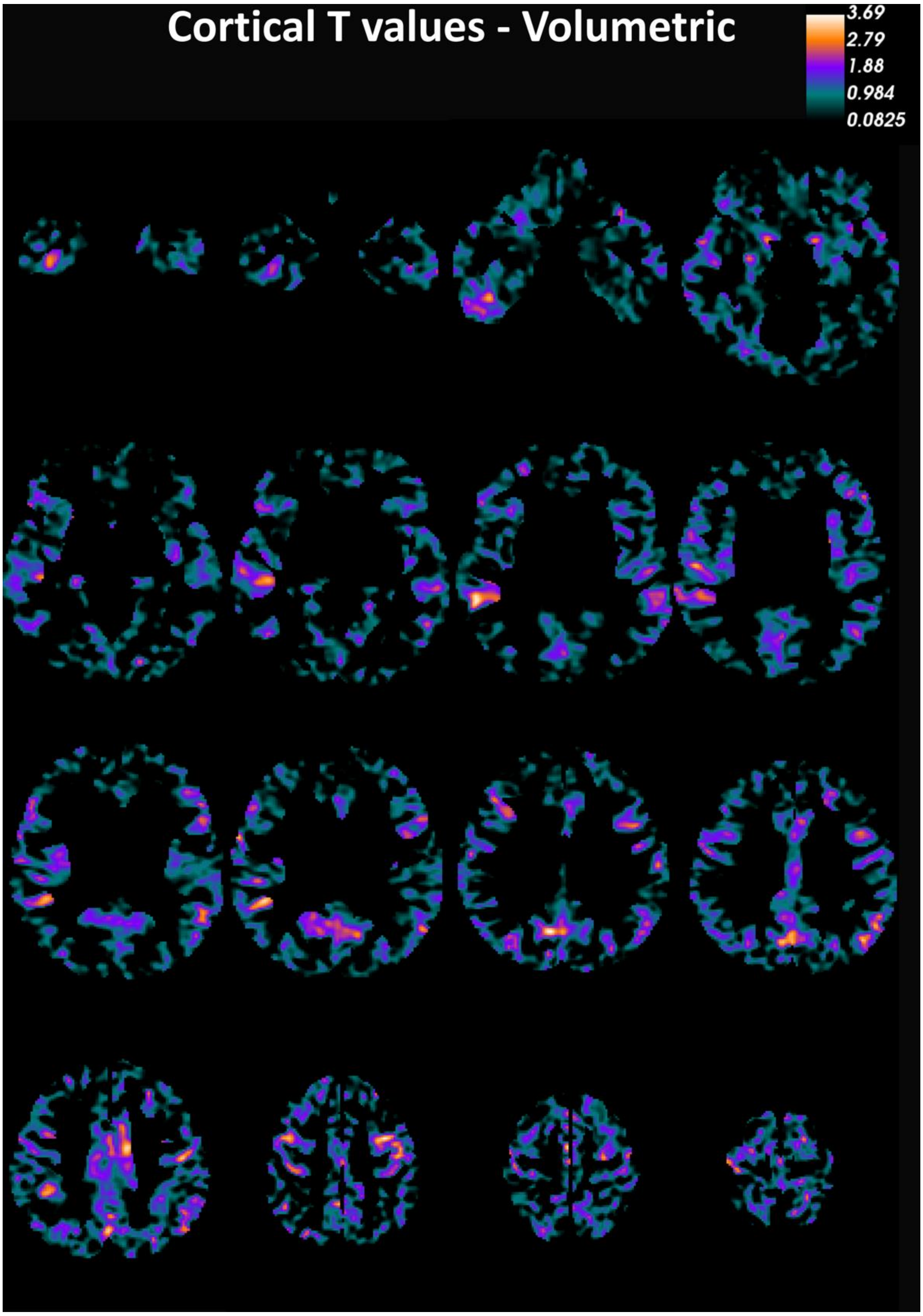

Figure 2 Map depicting the cortical T values obtained from the mean diffusivity analysis comparing HD expansion carriers and controls.

The colorbar represents the magnitude of the T scores (white: black, higher: lower). The T values are unthresholded. HD, Huntington's disease

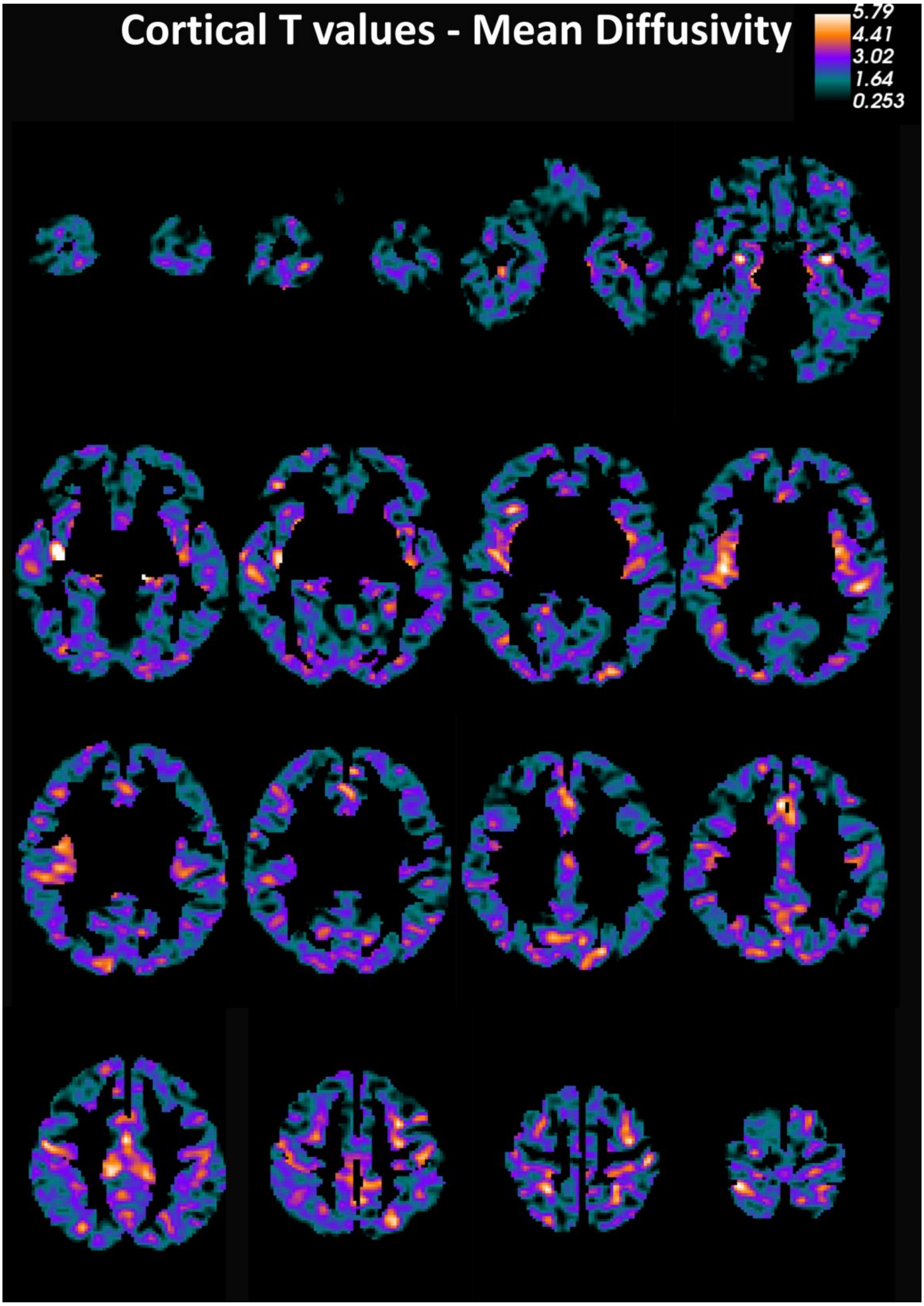
Cortical T values - Mean Diffusivity
5.79
4.41
3.02
1.64
0.253

Figure 3 Density plots illustrating the distribution of T values from the volumetric (left) and mean diffusivity (right) analyses comparing HD expansion carriers and controls

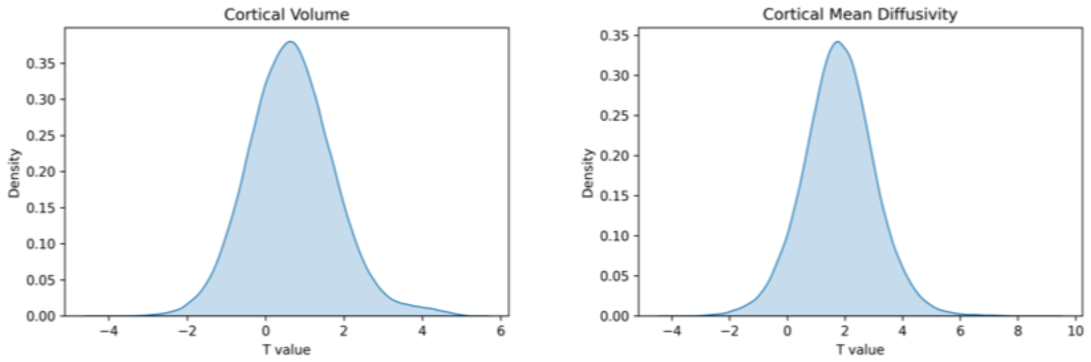

Figure 4 Summary of the analysis between HTT expression and developmental genes

A set of ten genes known to be differentially expressed during early cortical development was obtained from Kang et al. (Nature, 2011). The gene expression data for *HTT* and developmental genes was extracted from the AHBA across 110 regions of interest (ROIs), including cortical, subcortical (Desikan), and cerebellar (Diedrichsen) regions, using the abagen toolbox.
A principal component analysis was performed in the expression of developmental genes across ROIs. The first principal component was correlated with the expression of *HTT* across ROIs. Significance was determined using the P-spin test and through bootstrapping.

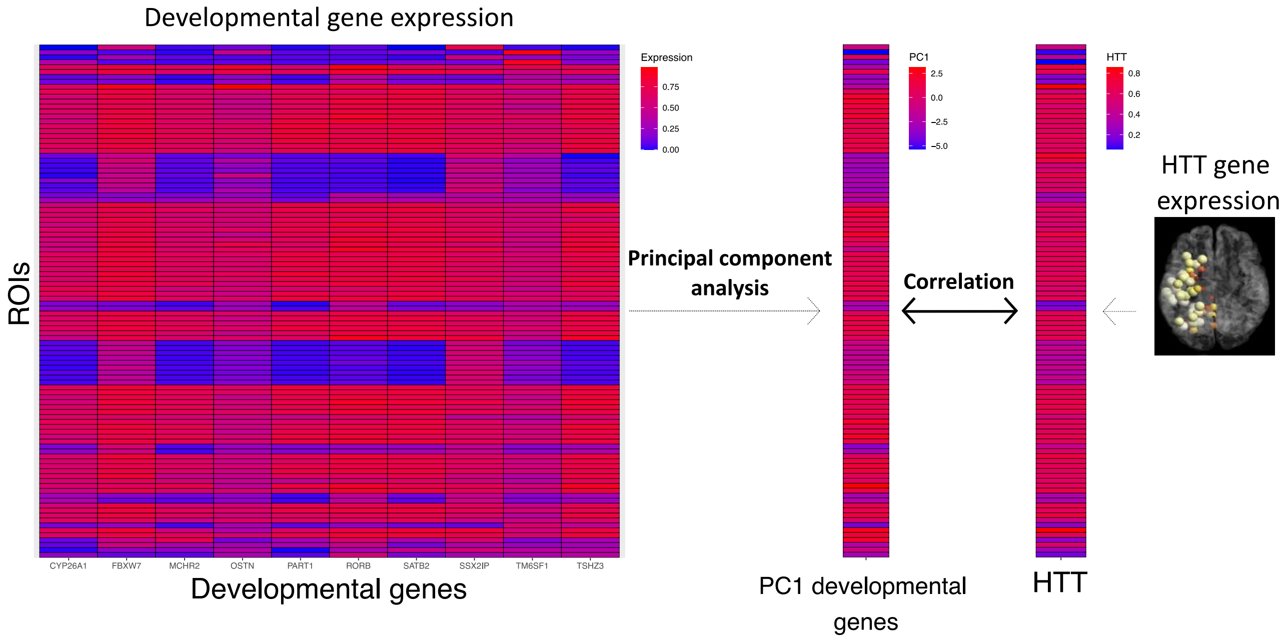

Table 1 Top 10% genes positively associated with cortical volume

Gene	T
GLI1	3.88580107
HIST1H3H	3.39847815
FMO2	3.26389359
ITGA11	3.2124481
BGN	3.15524776
PTGER1	3.0864971
SNAI2	3.0674731
ID3	2.9616936
ACO1	2.95740139
PMAIP1	2.91425275
GINS2	2.90816321
PECAM1	2.89622113
NPY2R	2.88353433
VASH2	2.88066286
TRPS1	2.85790295
TRAF3IP2	2.84642471
BMP6	2.8111231
PPP1R3B	2.8037253
TM4SF18	2.76617085
CXCR4	2.6840766
FAM117A	2.68270284
GPR182	2.68246512
CDH23	2.60900539
PDLIM4	2.59084608
NFIA	2.58738402
HAP1	2.57256284
TIMP3	2.56715779
APOLD1	2.56564361
PERP	2.56535708
PLOD2	2.54054722
FOXC2	2.53534146
NRN1L	2.53463101
NUP188	2.52810064
CYYR1	2.49024649
ME1	2.48732351
THBS4	2.46920629
PTGES	2.45477841
FOXO1	2.44808126
VANGL1	2.44791166
GBE1	2.43408656

PLEKHA7	2.40271812
THSD4	2.39768845
TMEM215	2.36333168
MATN3	2.34909877
GPR39	2.34649896
IL7R	2.33829496
C22orf15	2.33495334
FAM181A	2.33470644
HDAC7	2.33457572
SLC39A8	2.32911191
GALR3	2.32722194
PARP10	2.32363115
NINL	2.31362078
KLHL1	2.29934257
TRIL	2.29901214
GABRE	2.28803047
AVPR2	2.2774752
VANGL2	2.27520983
RAB33B	2.26582196
CCDC6	2.26446436
PDE11A	2.26005054
ITGA6	2.25996385
EPB41L4A	2.25142763
PELI2	2.24852174
ZNRF3	2.24634436
SPATA18	2.2391544
LAPTM4A	2.2323171
DNAH12	2.22917396
COMP	2.22650656
GLUL	2.21962672
HPR	2.21754523
HLA-G	2.21524752
AQP5	2.21524528
RAET1K	2.20745743
ZSCAN1	2.20187657
CRB1	2.19903604
SYPL2	2.1890825
TUBB2B	2.18555866
AIRE	2.18352414
CDSN	2.18257181
TBX19	2.17964303
ZIC3	2.15402555
OR5B17	2.1479751

TIE1	2.14376036
ACR	2.14094043
AEBP1	2.1378673
LFNG	2.13534109
KLHL13	2.13392929
MGST2	2.12516246
LYPD2	2.12487237
IRGC	2.12149359
CTGF	2.1194305
PLAGL2	2.11313983
C11orf16	2.11184703
EPCAM	2.11009803
SLC7A10	2.10443007
PPP1R13L	2.09981952
USP49	2.09704019
MYCT1	2.0938603
CA5A	2.08752301
S1PR1	2.08723955
HMGCR	2.08572608
SYDE1	2.08414121
S100A4	2.07207849
PRKCH	2.0707388
GPR4	2.06958381
PXDN	2.06945497
SLC26A2	2.06704051
CDR1	2.06370601
LOXL1	2.06170005
PIRT	2.05539544
LRRTM2	2.05428883
PPIC	2.05116533
TP53INP1	2.0510653
ITGA9	2.0496379
PPBP	2.04459272
MYOD1	2.039352
GBP2	2.0369293
GPBAR1	2.0355497
LRIG1	2.03257702
IGFN1	2.03002517
HSD3B7	2.02507047
SYNPR	2.02207339
ITGA1	2.02168711
MICA	2.01996738
EDN3	2.01933677

PLSCR4	2.01908473
WT1-AS	2.01650593
ERG	2.01373598
SLITRK6	2.00843479
FSTL5	2.00424786
MED25	1.99920916
LAG3	1.99844612
MYH11	1.99543516
OR52W1	1.99371016
TMED10P1	1.99076191
TTC36	1.989561
WFDC12	1.98945955
CXCR3	1.98889918
MYEOV	1.98858878
ITGB4	1.98852782
PGAM2	1.98569344
SMO	1.9849048
BHMT2	1.98199314
SLC38A5	1.97819895
C1orf226	1.9754995
CLDN14	1.97491332
FKBP10	1.97447818
SLC38A2	1.9712322
SNX33	1.96977071
CBR3	1.968428
HTRA3	1.9622445
SLC2A10	1.96092165
SLC22A31	1.9606276
KCNK7	1.95134379
TGIF1	1.94696713
LALBA	1.94615627
CRB3	1.94567124
PRRX2	1.93776174
ZSCAN22	1.93619924
ITGA7	1.93452595
N4BP2	1.93329367
AMN	1.93208044
SLC25A20	1.92966746
RCOR2	1.92959624
MLPH	1.92555451
PRX	1.92260883
NCF4	1.92069507
LRRN1	1.91780997

FAM201A	1.91631036
GRP	1.91629273
TRIM71	1.91563104
TTPA	1.9148975
PBXIP1	1.91311339
NPW	1.90461061
SRPX	1.90275115
CCT5	1.89907087
GPR153	1.89691028
CGNL1	1.89243495
INSIG1	1.89230976
BTN3A3	1.89188584
SPRR1A	1.88850588
IFIH1	1.88160627
NXNL2	1.8781233
LOC441204	1.87797139
HEYL	1.87634381
SSX5	1.87445228
FOXD3	1.87444365
IFIT3	1.87253933
SLC4A4	1.86856036
MTTP	1.8654234
PLCB4	1.86458691
CPEB3	1.86364234
SERPINH1	1.85981342
CHST7	1.85969983
RNF135	1.85549291
DACT1	1.85351138
MOV10	1.85298491
PCMTD2	1.85168968
KLHDC10	1.85168923
SBF1P1	1.84874777
NTSR2	1.84243373
FBXO46	1.84215003
C1QL2	1.84192103
SLC22A5	1.84186478
LRCH2	1.84163211
RHOD	1.84162936
WFDC8	1.83974795
LCA5	1.83785919
EVX1	1.83775372
BMP1	1.83398955
PTN	1.8339746

TMEM40	1.83224115
MCM3AP-AS1	1.83061237
MSLN	1.82992813
CPVL	1.82970201
HLA-F	1.8260819
SPINK8	1.82356548
ZNF843	1.82270366
MMP14	1.82212925
CTF1	1.82144408
CASKIN1	1.82090236
CDH6	1.8198467
HIST1H3B	1.81869053
TMEM72	1.81774457
IGFBP7	1.81635135
DERA	1.81564731
ANXA1	1.81523378
CRCT1	1.81266626
PRRX1	1.81246481
DHH	1.81097889
OR5H1	1.80819897
GBP3	1.80611047
CARD10	1.80469638
GP9	1.80388743
BEST3	1.79956138
HOXA3	1.79872925
PTH2	1.79671494
FAM84B	1.79617956
PI3	1.79603274
PAX8	1.7928923
ADORA2B	1.79281805
HS3ST5	1.79223807
NFIB	1.79189092
PLEKHA4	1.78957282
APLNR	1.78876088
PDGFC	1.78790743
ALS2CL	1.78670707
RBPMS2	1.78570878
IDH1	1.78522671
LGR6	1.78349776
TPSG1	1.78180475
SMCR8	1.77912863
IGF2BP2	1.77893752

SP7	1.77776256
FGR	1.77553796
GCLM	1.77456628
COL7A1	1.77431829
MXRA8	1.77112888
PBX4	1.76874206
PHYHD1	1.76744834
SLC9A9	1.76686527
PMEPA1	1.76633549
SOX18	1.76608965
HPDL	1.76507558
ANP32E	1.76238433
NHSL1	1.76160322
TERT	1.75999271
IVL	1.7597108
KRTAP12-1	1.75824315
TMEM88B	1.75813577
THSD1	1.75763209
SALL2	1.75658035
ZBED4	1.75652844
EPHX2	1.75457998
PCSK9	1.75353906
TNC	1.75104736
ADAMTS2	1.74897918
ALAS2	1.74616793
PLBD1	1.74533932
KIF26A	1.7414113
SOX8	1.74104118
ODF3L1	1.74003394
TAS2R39	1.73940922
PRKX	1.73888121
LCE1F	1.7385824
ENTPD2	1.7384396
ADCY4	1.73815302
EFCAB6	1.73751852
SCGB3A1	1.73696595
OLFML2A	1.73544255
TFF3	1.73401257
DCHS1	1.73128843
MYD88	1.73027822
GAB4	1.72603352
SRP72	1.72551573
PDGFRB	1.72391867

MAP3K2	1.72386049
MGAT4C	1.72215846
FAM129B	1.71991754
LRRC17	1.71968286
CXCL2	1.71898237
IFI27	1.71865916
TES	1.71855303
MICALL1	1.71763489
FAM43A	1.716894
KLK2	1.71679224
SLC16A8	1.71315884
SLCO2A1	1.71263707
ZNF274	1.71161213
PRDM6	1.71127779
KRTAP5-10	1.70945038
ACAA2	1.70880965
DDR2	1.70868924
SLC44A2	1.70856686
KDSR	1.70801075
GJB3	1.70782356
ITM2C	1.70757391
ADARB2-AS1	1.70739892
SASH1	1.70698142
KRTAP20-2	1.70525861
CEP170	1.70481233
EIF3M	1.70403822
LYPD1	1.70193046
RGR	1.7018399
CPNE3	1.70103748
KRTAP10-9	1.70063113
DKK4	1.6994777
APOA1	1.69772392
PON2	1.69397514
FXN	1.69334962
LY9	1.69287641
LINC00475	1.69251296
ABCB1	1.6912022
KCNJ16	1.68957346
GRIK4	1.6886129
MGC70870	1.68677068
PLTP	1.68590171
GPR63	1.68539826

GNA13	1.68507464
LCE3A	1.68136641
LAMB2	1.67917282
OXER1	1.67760521
SYCP2	1.67702123
ARL4A	1.67630127
IRX4	1.67614539
KCNIP2	1.67366191
RBM43	1.67280252
ITPRIP	1.67038775
GANAB	1.67022886
NEUROG1	1.6698939
SLC7A5	1.66947425
HLA-L	1.6693273
ATP4A	1.66885171
CRYL1	1.66799988
FFAR1	1.66587188
MRVI1	1.66581502
TTN	1.66292282
RETN	1.66254883
NPTX1	1.6596038
RGMB	1.65358193
HGF	1.6528458
HLA-DQB2	1.65280975
PLA2G15	1.64993604
ALS2CR12	1.64579507
ATP7A	1.64514238
GLUD1	1.64414642
TOP1P2	1.64284231
GNG11	1.64132685
NLRC3	1.64089089
CADM2	1.64068866
ARTN	1.63945431
PHF21B	1.63862014
C10orf99	1.63842157
ACP5	1.63719851
H6PD	1.63498889
GH2	1.63404681
GPR157	1.63131855
LHFPL5	1.63025548
PTGIR	1.62951425
ARID4A	1.62941849
C6orf201	1.62897482

BTN3A2	1.62818115
ACTA2	1.6276856
HEG1	1.6275141
TMEM100	1.62738181
HSD17B7	1.6270911
OTX1	1.62699228
MTMR9LP	1.62662158
KIAA1586	1.62649152
TSPAN10	1.62643847
ATP2C2	1.62475436
PALMD	1.62446382
PTGR1	1.62443512
ACTL6A	1.62307494
COL18A1	1.62302814
ABCG2	1.62301659
ATG4A	1.62199
POLE2	1.61943394
CLASP1	1.61769914
DNAH2	1.61549543
ADAD2	1.61544047
PIGA	1.61536315
SPIN4	1.61518957
MED17	1.61498737
SECTM1	1.61429387
PHOX2A	1.61401249
PCOLCE	1.61293905
C16orf92	1.61290203
PIPOX	1.61253823
ATP8B3	1.61181064
ADAMTS9	1.61168329
GNB3	1.61137177
CYTL1	1.60979194
KISS1	1.60852537
PTPN18	1.60849145
GOLIM4	1.60768604
SBK2	1.60747361
CYP11A1	1.60599238
ANTXR2	1.60588241
GDPD2	1.60465658
IRF2	1.60344927
ANKRD18A	1.60040368
LMAN1L	1.5999479
TMBIM4	1.59958959

ANKRD33	1.59871304
SDK1	1.59592171
TUBA3C	1.5946917
F2R	1.59421627
GLCCI1	1.59367367
KRTAP12-4	1.59317274
SLC12A4	1.59297899
PHLPP1	1.59286169
DDX58	1.59237458
COL16A1	1.59217361
ABCB7	1.59207904
ACP6	1.58969156
CNR1	1.58926709
EPHA8	1.58861088
UCN2	1.58826416
IFITM5	1.58780211
DMGDH	1.58773131
PSORS1C2	1.58649099
NXF3	1.58522662
DPT	1.58202726
AR	1.58132742
CD44	1.58093265
PLAC8L1	1.58075863
Mar-03	1.57925391
MYCN	1.57880575
CPAMD8	1.57874355
KRTAP5-11	1.57794051
NFATC1	1.57728216
TMEM52	1.57716591
SEMA4A	1.57705037
GOLPH3L	1.57672781
KRT19	1.57629223
C5orf22	1.5758246
SOX13	1.57540123
HSPA1A	1.57464874
RNF152	1.57423979
RYR1	1.57223851
TBX5	1.57169997
CLPS	1.57010199
ZG16B	1.5698743
ARL4D	1.56914737
ZMIZ1	1.56909268
BAHD1	1.56702092

SOX4	1.56575696
ADPGK	1.5656415
NKX2-6	1.56508377
USP37	1.56454582
ITM2A	1.56152957
THUMPD1	1.56086404
PLG	1.55965357
COL1A2	1.55944845
EDNRB	1.55835177
ITM2B	1.55772622
EMCN	1.55653596
FOXL2	1.55651903
CC2D2A	1.5540975
SOX17	1.55309245
SLC7A11	1.55273932
SEMA3A	1.55194612
AMZ1	1.54989127
MT1L	1.54918778
AQP6	1.54907058
FOXC1	1.54895195
SFT2D3	1.54882637
DCST2	1.54838844
S1PR4	1.54726579
SPRYD4	1.54700818
KRTAP10-5	1.54664758
DLK2	1.54539967
ITPR3	1.54352031
FBLN2	1.54178314
STAG2	1.54013606
KRTAP8-1	1.5401033
UPK3A	1.53998619
GOLGA8A	1.53934894
BEND7	1.53900115
MAP2K3	1.5384765
NET1	1.53829639
ZAN	1.53599658
MSX1	1.5350488
CLDN5	1.53384251
SGK494	1.53360399
OR5V1	1.53276612
SYNPO2L	1.53265263
DBI	1.53215332
CXCL14	1.53171232

CRYBB3	1.53157725
A4GALT	1.53114951
GKN1	1.53003677
ZBTB12	1.52999848
GPR25	1.52988752
MAOA	1.52726369
KDM5B	1.52570545
FGF3	1.52553302
CSRP2	1.52517202
THBS1	1.52484008
A2M	1.52472133
TBCCD1	1.52471024
PEX11A	1.52462312
COL6A3	1.52359249
GIMAP7	1.52287353
ZNF208	1.52273424
ECSCR	1.52265934
SQLE	1.521965
KRTAP6-1	1.52075795
DNMBP	1.5203733
GNRH2	1.51873967
SOD3	1.51834972
CLCN1	1.5167321
ZNF596	1.51623327
NFRKB	1.51608616
IER5	1.51602392
REM2	1.51563842
BMPR2	1.51538243
PDE6G	1.51494807
PLVAP	1.51412229
ABCB6	1.5134354
FEM1A	1.51343174
CAMK2G	1.51329943
CDH16	1.51324896
SCRT2	1.51297481
ZNF784	1.51133595
SERINC1	1.51128986
LIX1	1.51065595
BTNL2	1.50948568
ART3	1.5055337
DCAF12L2	1.50375826
NXT2	1.50370623
KLF15	1.50309133

KRTAP10-3	1.50282828
UG0898H09	1.50274304
AQP2	1.50269611
KRTAP19-1	1.50268799
LOC728024	1.50224944
HES5	1.50140092
FAM83E	1.50094254
HSD11B1	1.49971704
GPR37L1	1.49870052
RASL12	1.49675874
SPRR2C	1.4966677
AGT	1.49651257
RPS6KA6	1.49574661
SLC1A3	1.495367
DEFB115	1.49168366
MT1B	1.49158473
SENP6	1.49126278
P2RX3	1.49019498
OR1E1	1.48997699
RAB34	1.48955151
TCF15	1.48899978
KDM5A	1.4887226
ID4	1.48859161
COX6B2	1.48836381
KRTAP21-1	1.48780274
EIF3IP1	1.48745866
NAGS	1.48661299
PTCD3	1.48565866
LINC00304	1.48262356
NCAPG2	1.48262017
NEK8	1.48256889
KRTAP2-1	1.481509
GPAM	1.48123907
RTN4RL2	1.48095916
VAX2	1.48092235
SLC44A3	1.48056822
WNT11	1.47985091
ISM2	1.47783488
ARSK	1.47703213
ISLR2	1.475702
ZC3HAV1L	1.47368698
MYL3	1.47332286
ZSWIM5	1.47287257

MYOG	1.47242601
ZFP36L2	1.47191025
CKMT2	1.47097046
CAV1	1.46995112
SLC2A4RG	1.46960535
DARS	1.46957289
PDYN	1.46909135
FAM47C	1.46776796
CD81	1.46748698
ACTG2	1.46715824
CD27	1.46703987
TRIM49	1.46556306
GTF3C4	1.46549248
TMEM30B	1.46429631
OR10H2	1.46074202
TMEM150A	1.46071478
MAMSTR	1.46025823
BMP2	1.45984885
SERPINE2	1.4594558
REC8	1.45934062
GSTM3	1.45855166
DGKG	1.45854496
MUC2	1.45820001
SHANK2-AS3	1.45722475
SPNS3	1.45616948
KRTAP5-3	1.45614901
FGFRL1	1.45521095
SLC1A2	1.45516571
KCNIP1	1.45373429
ZNF648	1.45346237
KBTBD6	1.45320318
CCR3	1.45211803
MYADML	1.45205326
BARX1	1.45152948
GNLY	1.45115748
ZNF229	1.44912347
NPC2	1.44718734
TTC38	1.44649467
ELN	1.44643263
GEM	1.44561553
BTBD17	1.44530524
EIF2AK3	1.44435164

LAMA1	1.44340171
CD109	1.44322798
MMP9	1.44302021
GPR152	1.44246749
ASAP3	1.44229748
PPP1R1A	1.44192169
CHST14	1.44097037
MT1X	1.44090391
BCL11B	1.4402234
RBM12	1.439246
CDK4	1.43820658
GCK	1.43780099
KRTAP20-1	1.43604044
HPSE2	1.43517207
DNMT3B	1.43455793
SLFN11	1.43443476
ST7-AS1	1.43440608
STK33	1.43394678
RRN3	1.43385748
ZFHX4	1.43380117
BLCAP	1.43369846
PDCD5	1.43322291
EFNB1	1.43307396
PDLIM5	1.43302106
ULBP1	1.43143349
PDP1	1.43056733
SCG2	1.42923835
LY6G5C	1.42803728
LEPROT	1.42635517
FAM167A	1.42558407
XCR1	1.42501923
CALCA	1.42424975
NLRC5	1.42185501
IRX3	1.42181264
LMO2	1.42169888
CCR9	1.42110144
BNIPL	1.41907105
PPARA	1.41836638
NPAS3	1.4170174
TMEM120B	1.41629326
LCE2D	1.41622794
BIRC3	1.4160589
OR2Z1	1.41590257

TNFRSF12A	1.41486989
PLRG1	1.41483204
ITGAV	1.41455424
UHRF1	1.41399719

Table 2 Top 10% genes negatively associated with cortical volume

Gene	T
PPM1L	-3.29551384
GRB14	-3.24792976
COL10A1	-3.10444295
FAM162B	-3.08467349
NELL1	-2.87059658
AMACR	-2.8461087
BAIAP2L2	-2.74602815
PKD2L1	-2.66747213
CDK5R1	-2.64009071
COL5A2	-2.62711306
MRPS30	-2.61976925
KRT83	-2.61765496
C1QA	-2.58965417
SCN8A	-2.4975413
RPS19BP1	-2.48523945
LRRC6	-2.48327837
NUDT14	-2.48013273
LCN15	-2.47661674
PTTG1	-2.4711451
ZNF717	-2.46440991
SLC36A1	-2.44312456
TTR	-2.41185023
FAM49A	-2.39116565
FAM182B	-2.37808979
TMEM65	-2.36718555
RPS24	-2.34676723
CDT1	-2.34534546
COL19A1	-2.34274626
PHLDA2	-2.32332855
CAMK4	-2.32054714
UST	-2.30227162
NOV	-2.30002486
COL24A1	-2.29010284
ZNRF2	-2.27623914
TRAPPC1	-2.27431928
RASGRF1	-2.26516357
C1QB	-2.26257398
ISG15	-2.25266104
DBNDD1	-2.24294098
SHROOM3	-2.23809376

RARB	-2.23261251
PTTG2	-2.21600345
SLC2A5	-2.21341536
FGF17	-2.21244588
TYROBP	-2.18208791
RDH12	-2.1791099
ENTPD4	-2.16246471
SIGLEC14	-2.15573022
CRYBB2P1	-2.14569079
PPIAL4G	-2.13165139
CARTPT	-2.12139593
SAMD14	-2.11295936
RPS21	-2.11199667
OLFM3	-2.10999371
GABRB2	-2.10507687
USE1	-2.10166197
FSD1	-2.09275093
RAB27B	-2.09073084
ASGR2	-2.08951546
FAM78B	-2.08617787
TPK1	-2.06964817
KCNH5	-2.06767649
CCDC148	-2.06372555
TRAF3IP3	-2.0560192
RAB11FIP5	-2.05412997
PPL	-2.04210485
PPEF1	-2.04191896
GRIN2B	-2.04008312
TM6SF1	-2.03647626
SPTSSB	-2.03392426
MET	-2.03185339
HSPB3	-2.03086187
MAP3K12	-2.02995551
TMEM191A	-2.02776914
HOXB3	-2.0275512
FAM20A	-2.02594076
RASSF7	-2.02395222
ADCY8	-2.0126444
FAM185A	-2.00741713
LRRC39	-2.00586937
CX3CR1	-2.00518815
DCBLD1	-2.00410412
BDKRB2	-2.00118939

MARCH11	-2.00052194
NXPH2	-1.99805764
RPS29	-1.99228617
CSF2RA	-1.98956576
TMEM141	-1.98013952
STAT4	-1.97783456
ZRANB3	-1.97349053
EXOSC1	-1.97077992
CAMK2D	-1.96893501
NAPSB	-1.96872454
POLD4	-1.96528072
PCDHB2	-1.96181663
PELI3	-1.95685049
SIDT1	-1.94582477
ARHGAP26	-1.94472913
OSTF1	-1.94464034
EPHA6	-1.93770694
ARHGDIG	-1.93619088
DPYSL4	-1.93095422
SATL1	-1.93059377
NTNG1	-1.92757207
ROGDI	-1.92653834
ADCY1	-1.92580833
CBX4	-1.92409886
INO80E	-1.92142354
NIPA1	-1.9201265
ASIP	-1.91337926
WFDC2	-1.90604248
TLN2	-1.90383831
NT5DC3	-1.90052122
FAR2	-1.90020631
TRPV3	-1.89786404
NEUROD6	-1.894338
TMEM160	-1.88957342
ALKBH6	-1.88692943
SUSD3	-1.88315649
PAPSS2	-1.88314614
C11orf24	-1.8817671
COX4I2	-1.88057265
RAB15	-1.87769944
PAK1	-1.87682644
ZDHHC13	-1.87678632
ANAPC2	-1.87128249

RPH3A	-1.87021013
GTPBP10	-1.86970167
ARL15	-1.86606592
SERPINI1	-1.86494774
SPRN	-1.85648852
GLMN	-1.8547508
IP6K1	-1.8504207
SDR16C5	-1.8496433
BEND5	-1.84923991
SUSD5	-1.84440547
P2RY12	-1.84215602
ARMC7	-1.83961509
DLEU7	-1.83900236
SYCE1	-1.83879436
CDCA8	-1.83381206
OSBPL10	-1.82763942
POLR2I	-1.82597102
PPP2R2C	-1.82394492
ZBBX	-1.82336262
SCAMP5	-1.82232731
NHP2	-1.8198052
GNPAT	-1.81953658
PPP5C	-1.81761303
TLL1	-1.81652029
THRB	-1.81253721
WDR27	-1.81157451
MYO1B	-1.81035172
GTF2I	-1.80858316
KIF17	-1.80695013
PDK3	-1.80634725
NCAPH	-1.80380763
KCNB1	-1.80189332
MRPS35	-1.79908313
NAA50	-1.79326296
ZMAT5	-1.79318808
BAD	-1.79312141
SERP2	-1.79015633
KCNIP3	-1.78871057
CCDC122	-1.78017023
ME3	-1.77957807
STAP2	-1.77643508
IAH1	-1.77292178
LOC728743	-1.77076688

PANX1	-1.7702668
MYO1F	-1.76887791
C12orf10	-1.76829405
DYNLL2	-1.76759947
CCDC87	-1.75937147
MANSC1	-1.75800472
MAST1	-1.75726859
SSH2	-1.75473085
RAC3	-1.75172203
MRPL3	-1.74811982
AKTIP	-1.74695822
UQCRQ	-1.74538468
ZBTB17	-1.74427873
MTMR4	-1.74397693
VWC2L	-1.74373237
CNTNAP5	-1.74227974
PPM1J	-1.74009158
FAM98C	-1.7369151
COBLL1	-1.73660201
TTBK2	-1.73162366
PTPN3	-1.73058237
COX8A	-1.72770805
PGP	-1.72695732
ALOX5AP	-1.72632147
MYBPHL	-1.72426531
NOP16	-1.72338711
GNG13	-1.72154644
CPLX1	-1.72119896
FREM3	-1.72076672
SGTB	-1.72010767
HIST1H2BD	-1.71943975
CHCHD5	-1.71776132
DPM2	-1.71608888
ETV6	-1.71555692
RHEBL1	-1.71548615
SERPINF1	-1.71541803
RASGEF1B	-1.71477209
HSPBP1	-1.71163782
OR2L3	-1.7072141
LST1	-1.70647047
RGS18	-1.7052355
ADRA1D	-1.70493624
NDUFV1	-1.70486232

PRKG2	-1.70477024
USF1	-1.69884828
CTXN3	-1.69819586
GNG3	-1.697092
TRMT12	-1.69563055
NLE1	-1.69231689
GPR34	-1.68515621
DTX4	-1.68259919
RTN2	-1.68229362
TSC22D1	-1.68100054
APRT	-1.67911682
KPNA5	-1.67266441
ARL9	-1.66804141
TMEM143	-1.6671669
IL26	-1.66598806
TIMM10	-1.66335968
KCNK12	-1.66251224
CHI3L1	-1.66138147
LAPTM5	-1.66053026
NPM2	-1.65999766
RRP9	-1.65958536
RGS10	-1.65930112
FDX1L	-1.65897028
TGFBRAP1	-1.6565792
VSIG4	-1.65553652
RASAL3	-1.65452795
B3GAT1	-1.6543154
MATK	-1.65415352
BRMS1	-1.65130787
ZNF711	-1.65101206
FBXW9	-1.65064911
CNTN5	-1.64738696
ADAMTS19	-1.64678323
TAF13	-1.64565538
POPDC3	-1.64563969
ERI2	-1.64375409
SATB1	-1.64296657
GOSR1	-1.64243595
ELOVL6	-1.63895121
MFSD3	-1.63053859
MRPL36	-1.62804909
PPP3R1	-1.62665695
PRKAR1B	-1.62576411

NAPB	-1.62315006
NCALD	-1.62292034
CCDC124	-1.6223927
GAB2	-1.618717
GSTZ1	-1.61805802
TAF8	-1.61767495
SORL1	-1.61447731
ACOT8	-1.60557288
FAM110A	-1.60306185
MGAT5	-1.59932311
EIF2B2	-1.5989508
CTSS	-1.59858512
UXS1	-1.59532453
TRAPPC9	-1.59367696
SMUG1	-1.59326145
XYLT1	-1.59245677
IQCJ	-1.59218387
ZCWPW2	-1.59161589
RIMS1	-1.59068913
PPFIA3	-1.5883477
MYRIP	-1.58808524
KCNV1	-1.58709513
OPN3	-1.58244844
RFC4	-1.57829253
DENND1C	-1.57685707
VPS25	-1.57661496
ZNF280A	-1.57527544
DDT	-1.57502782
HAMP	-1.57227061
HDC	-1.57136563
MRPS12	-1.57128257
TDRD7	-1.57111983
FAM69B	-1.56706976
SYT16	-1.56644618
LPAR1	-1.56533327
RTN4IP1	-1.56446135
PRSS16	-1.56388275
SLC22A18	-1.56323917
ARNTL2	-1.56275053
TSHZ2	-1.56031057
HS6ST3	-1.56013551
NUDT22	-1.55986205
UQCR11	-1.55947587

HCST	-1.55864198
RPL31P11	-1.55822408
C6orf62	-1.55566885
RNF214	-1.55385463
PIN1	-1.5533024
ZNF385B	-1.55304847
NSMCE2	-1.55239157
RPS27	-1.55130831
PIK3AP1	-1.55060517
PLCXD1	-1.54957711
PTPN4	-1.54924003
RPL36	-1.54833418
RAB3D	-1.5474649
FAM173A	-1.54713725
APP	-1.54562385
KCNH1	-1.54261929
NR2C2	-1.54251821
MPG	-1.54190129
PTPN21	-1.54156676
NUBP1	-1.53755284
ADAMTS5	-1.53643754
LY6G5B	-1.53327783
CBX3	-1.53326595
L3MBTL3	-1.5320461
XRCC4	-1.53032882
COX7B	-1.52884761
TEKT5	-1.52819071
TRMT61B	-1.52727235
EML1	-1.5255224
PPM1M	-1.5225343
AIF1	-1.52190571
DGKI	-1.52171939
MIEN1	-1.51932517
NDUFA1	-1.51896616
MAPK1	-1.51781909
SPHK2	-1.51766326
EXOSC5	-1.51561354
NAPEPLD	-1.51504626
OLR1	-1.51467765
TTLL7	-1.5143643
ZNF182	-1.51367542
ZNF668	-1.51304092
ATG12	-1.51101757

CCDC39	-1.51031055
GPR108	-1.50891131
HINT3	-1.50770685
CPNE7	-1.50563966
CCDC151	-1.50528408
CCM2	-1.50434504
GFM1	-1.50379199
ELAVL4	-1.50306129
MOCS3	-1.50297751
ABHD5	-1.50208074
RELL2	-1.50177789
ARG2	-1.50135024
C11orf80	-1.50052664
MIPEP	-1.49928735
WDR55	-1.49915792
FBXL15	-1.49853594
C12orf54	-1.49835894
MED13	-1.49605199
EXOSC4	-1.49389461
NDUFA13	-1.4929828
HGD	-1.49225733
TMEM232	-1.4903588
ZNF471	-1.49010867
PID1	-1.48894955
TNFRSF11A	-1.48844617
NANOS3	-1.48733502
CCDC107	-1.48711317
PITPNM1	-1.48672944
MRPL55	-1.48598653
FAT3	-1.4850951
MPV17	-1.48488031
CKMT1A	-1.48436158
RHOT1	-1.48421283
PMS2P1	-1.48288297
C12orf4	-1.47912225
ATP6V1G2	-1.47859018
STRBP	-1.47633993
DUSP4	-1.47572005
TMEM11	-1.47491739
MARCH2	-1.4747343
C19orf70	-1.47459956
ACTN4	-1.47300323
N6AMT1	-1.47289306

CD3EAP	-1.47205726
RPA3	-1.46986915
MPP6	-1.4690783
NT5DC2	-1.4688075
LAIR1	-1.46074046
CDH8	-1.46050438
MRPL18	-1.46023479
MPND	-1.45864129
DDX49	-1.45717424
NICN1	-1.45586049
DNAJA2	-1.45550315
SMYD5	-1.45548098
SLC25A10	-1.45509475
DTWD2	-1.45377742
TBC1D30	-1.45374954
ZFYVE9	-1.45308043
SNX10	-1.45169163
SLC25A46	-1.45133744
DDX41	-1.44980387
REXO4	-1.44961438
TFPT	-1.4490748
RGS7	-1.44856472
ST6GALNAC5	-1.44813898
GLIS1	-1.4463153
FRMD5	-1.44371675
GAS5	-1.44304614
WDR83OS	-1.44234287
APBA1	-1.44143248
HIST1H4K	-1.44116945
TMEM68	-1.43951031
ZSCAN23	-1.43847777
FAM89B	-1.43781374
CD53	-1.43748265
SULT4A1	-1.43613346
SNRPE	-1.43580408
TLR6	-1.43553881
EPHA3	-1.43539492
TPST2	-1.43535645
STX1A	-1.43523273
TMOD2	-1.43342664
GPR183	-1.43252823
CA10	-1.43221134

OXR1	-1.4308572
PTGES2	-1.43072907
FIG4	-1.43068268
DIS3L	-1.429308
GGH	-1.42899879
RANBP10	-1.4289654
PDZD4	-1.42851117
RPL23AP82	-1.42776879
ZNF780B	-1.42747627
C3	-1.42711816
APBB1IP	-1.4269186
KATNB1	-1.42687961
SLC35A4	-1.42369452
VASH1	-1.42266904
LOC731157	-1.42229135
ANAPC1	-1.42188501
RPE	-1.42188402
GNB1	-1.42154617
SAMHD1	-1.41750906
ALKBH7	-1.41625681
C19orf47	-1.41607586
MAP2K2	-1.41471505
CDC6	-1.41462192
AAK1	-1.41410543
GRM5	-1.4139411
GDF10	-1.41370265
RNF170	-1.41228334
ELK4	-1.41049183
ITGA2B	-1.41002511
MCM7	-1.40946415
POLR2E	-1.40918422
NUDT6	-1.40871784
LMBRD2	-1.4084675
BTBD6	-1.40746342
ARL6	-1.40550572
DUSP3	-1.40455813
KCNB2	-1.40455139
MRPL21	-1.40450605
PHLDA3	-1.40413036
NDUFB7	-1.40342138
FGL2	-1.40243685
MAP2K6	-1.40234515
KHDRBS2	-1.4010049

TRMU	-1.40083603
GRIN2A	-1.39955197
ILVBL	-1.39919181
NEDD8	-1.39852502
BARD1	-1.39837608
RANBP9	-1.39830352
LARP4B	-1.39699891
RNF220	-1.39675847
FKBP3	-1.39663574
HRASLS	-1.39611847
TTC23L	-1.39605044
NDUFV3	-1.39465396
COPE	-1.39447713
USP12	-1.39435047
VPS4A	-1.39330752
S100Z	-1.39319066
MAP4K2	-1.39295858
NKAIN2	-1.39295423
NTHL1	-1.39235319
GMPPB	-1.39042419
HECW2	-1.38985114
TRPM4	-1.3891795
SLC44A5	-1.38833604
RAD9A	-1.38819692
COASY	-1.38754509
HIST1H4J	-1.38734865
VCPIP1	-1.38641521
FIBP	-1.38630053
HDAC5	-1.38547611
TRNP1	-1.38486767
DUSP2	-1.38430425
VMO1	-1.38407761
CALM3	-1.38323437
DUSP6	-1.38228081
FCGR2A	-1.38098453
S100A8	-1.38076644
SSNA1	-1.37931261
EHBP1L1	-1.37900531
CATSPER2P1	-1.3788177
DCXR	-1.37715011
SLC38A6	-1.37690977
RPF2	-1.3748986

PRDX5	-1.37395876
ALKBH2	-1.37394421
ADAM9	-1.3722963
C1orf162	-1.37199545
PTPRT	-1.37181549
GABRA3	-1.37098084
CLEC2L	-1.37063986
RPL10	-1.36988081
TNFSF13B	-1.36774365
CEP152	-1.36769686
TSHZ3	-1.36746462
TMSB10	-1.36725239
MCHR2	-1.36697644
PARP6	-1.3668965
NOP14	-1.36570628
HOMER2	-1.36491208
CHRNA2	-1.36416593
CYP46A1	-1.36357897
SEMA7A	-1.36325439
THAP7	-1.3607321
TTL	-1.36036444
ZNF592	-1.35995758
VIPR2	-1.35952712
YTHDF3	-1.35913548
TFEC	-1.35875761
WDR13	-1.35653323
MYO19	-1.35529518
RHCE	-1.35504648
FBXW5	-1.35438585
ABHD8	-1.3521005
ZNF517	-1.3519152
SLC25A16	-1.3509842
RASGRF2	-1.34837451
HK2	-1.34646657
ANKRD52	-1.34604879
ZNHIT3	-1.34538715
KCNJ9	-1.34499118
MAL2	-1.34350877
B4GALT7	-1.34264858
PET100	-1.34101818
TMEM132C	-1.33855094
XPO7	-1.33828213
ST3GAL3	-1.33686377

FMN1	-1.33675613
GDA	-1.33637638
ADAM23	-1.33626413
CBWD1	-1.33618044
FLOT1	-1.33533631
CNTNAP2	-1.33524344
VPS37D	-1.33377979
HLA-DMB	-1.33370309
ZNF526	-1.33333045
RASSF5	-1.33168863
VPS37A	-1.33161097
MRPL14	-1.33147444
SUPT3H	-1.33034605
NNT	-1.32941331
ANKRD9	-1.32934135
TPI1P2	-1.32804678
RNF26	-1.32789188
SERINC5	-1.32693837
L3MBTL4	-1.32607482
GSS	-1.32483677
EZH2	-1.32378803
SPHKAP	-1.32372012
VMAC	-1.32294756
RNF14	-1.32234346
MAP3K9	-1.32222927
BTBD8	-1.32158503
AMIGO1	-1.32154472
IGFBP2	-1.32039731
ZNF492	-1.32013462
HNRNPH2	-1.3194771
CYTH2	-1.31857304
ANKRD13C	-1.31800162
TYRP1	-1.31763695
ITGB3BP	-1.31675017
PLXNA1	-1.31545242
ANKRD13D	-1.3139857
EVI2B	-1.31273828
TTTY15	-1.31172751
UQCRHL	-1.31125617
GUCY1A2	-1.30936106
CCDC144NL	-1.30904331
CLUL1	-1.30785949
ANK2	-1.30577473

PTPRS	-1.30492288
GNAQ	-1.30465128
DCTN2	-1.30370319
AIP	-1.30288319
PEX16	-1.3026195
ACCS	-1.30233995
MRPL45	-1.30212992
MCRS1	-1.30157791
RPS15A	-1.29950797
SLC4A8	-1.29939188
RASGRP1	-1.29802577
LRFN5	-1.29677099
RPS12	-1.29670227
HIST1H1A	-1.2966862
ECSIT	-1.29468745
ZNF415	-1.29435717
SAMD12	-1.29312707
LIN7B	-1.29147296
TOMM5	-1.29143706
TUBA4B	-1.2884079
SPATS2	-1.28823816
ATG7	-1.28738065
PSMD13	-1.28664882
HLA-DRA	-1.2863059
ZNF740	-1.28504969
IFI30	-1.28438183
HARS	-1.28413663
TPD52	-1.28364275
L2HGDH	-1.2829018
PDCL3	-1.282151
CD74	-1.28197275
PIN4	-1.28137354
CSAD	-1.28077439
ASGR1	-1.28034047
WDR86	-1.28000263

Table 3 Top 10% genes positively associated with cortical mean diffusivity

Gene	T
MYH11	5.12758892
TMEM215	4.68741316
PECAM1	4.41648166
ACTG2	4.08416666
NXN	4.0341803
TAGLN	4.02089969
FMO2	4.01248038
HSPA1A	4.00106707
TPM2	3.99674603
DACT1	3.94604664
S100A4	3.93058643
CHRNA6	3.92851395
RELN	3.88172712
HSPB1	3.79896172
SUSD2	3.77971486
COL1A2	3.7498961
CNN2	3.71668479
AEBP1	3.60662833
PDGFD	3.57324785
REC8	3.55656092
HIST1H1D	3.53935961
ITGA7	3.515037
FAM129A	3.49063133
MXRA8	3.47878559
NUPR1	3.46994599
EPCAM	3.37719409
SERPINH1	3.31547044
MYL9	3.26755235
HSD11B1	3.25884554
FLNA	3.20979134
PLN	3.20433891
SLITRK6	3.20059801
DNMT3B	3.15088819
ACTA2	3.14014535
SLC2A12	3.12535372
GLI1	3.12524908
HMGN3	3.1094747
PGAM2	3.09160056
PTCH1	3.0711803
CXCL2	3.06969929
CRIP1	3.04748016

PHKA2	3.03207495
SCN7A	3.02086388
CD44	2.99793781
CD55	2.95154979
LMAN2L	2.95055276
APLNR	2.95041436
SRGAP1	2.91972214
PBX4	2.90999714
BTG3	2.90709745
OLFML2A	2.90671971
IGF2BP2	2.89351374
CLCA4	2.88942107
TOB1	2.88490586
MATN2	2.84831195
ACO1	2.84477082
ANTXR2	2.83418584
SPARC	2.81317311
PLXNB3	2.81004269
IQGAP1	2.80680644
RGS9	2.8055659
KRTAP12-1	2.79236935
MYH15	2.78641133
ABHD1	2.78521365
TRPC4	2.77764663
NFE2L3	2.77249738
RCN3	2.76977722
ANGPT1	2.75349288
AVIL	2.74470122
NPNT	2.74252132
ENOSF1	2.72951565
ITGB4	2.71818492
MGAT4C	2.71792571
DNAJB1	2.71700562
MMP14	2.71249211
PIRT	2.70941453
CLEC18B	2.69843631
TBC1D26	2.67639985
CDH23	2.6577128
TEKT2	2.65748251
CTGF	2.65369112
FOXO1	2.65342167
LYPD1	2.64662039
TBL1X	2.64655904

LIPC	2.63696294
RGS8	2.63403996
SLCO2A1	2.62808752
CMKLR1	2.626923
FANCL	2.62545341
MPST	2.60767448
GBP2	2.60742682
RSPH9	2.59235863
PON3	2.5864104
CYP2J2	2.5846482
DGKG	2.57488014
KLF4	2.57434751
ACTL6A	2.56577998
EGR4	2.56391186
ATF3	2.56105598
ALDH3B1	2.55860187
VWF	2.55232767
C11orf96	2.54957592
LOC554206	2.53661845
PDE5A	2.53475915
COL16A1	2.53021705
PDLIM4	2.50687883
HTRA3	2.50623596
TBC1D10C	2.50200452
RFX4	2.4940321
TCAP	2.48495149
PELI2	2.47903264
FOS	2.4764316
ANKRD45	2.46244616
PCDHGB4	2.4594932
FAM71F1	2.45042863
CAV1	2.44843359
FAM196A	2.44554284
ARSE	2.44510979
CCL19	2.44391566
C1QTNF6	2.44122275
CCDC154	2.43556593
MT1F	2.42888131
CXCR4	2.42607502
DLEC1	2.41435055
CCDC152	2.41196732
PEG10	2.40993231
B3GAT2	2.40954078

CDH2	2.396584
TMEM39A	2.39288893
NEXN	2.38992232
IL15	2.38866497
NINL	2.38740081
VANGL2	2.38214297
PPP2R1B	2.37204962
ADAMTS1	2.36959817
SP1	2.36813181
TP53INP1	2.36412902
SGSH	2.36029885
ZNF735	2.35640972
COL4A1	2.35403436
MICALL1	2.3510238
YY2	2.34734225
NDUFS7	2.34316978
LXN	2.3430193
TNRC6C	2.34197088
CPAMD8	2.33496891
AASS	2.33096442
TIMP3	2.32410092
ZNRF3	2.31959789
ADAM15	2.30446081
SSBP2	2.30293845
ASB2	2.30126938
GRPR	2.30058344
OAS3	2.29637425
CDK4	2.29265865
SREBF1	2.29226628
PYGO1	2.29050874
EMP3	2.28935091
SLC4A11	2.28443944
ZNF774	2.28040455
DOCK6	2.27542592
ITGA8	2.27220021
SLC7A2	2.27053094
FIBIN	2.26862204
CALCRL	2.26675545
EMP1	2.26224348
PTTG1IP	2.26120133
PIGA	2.2570553
HSD3B7	2.25319445
STK33	2.24740741

HLA-G	2.24404963
CXorf57	2.24257146
SPRYD4	2.24022351
DNA2	2.23918431
GLUD1	2.22973387
PLBD1	2.22523853
PABPC1L	2.2210071
CFB	2.2189998
DOK1	2.21299293
LAMB2	2.20785302
SLC30A1	2.19820485
LOC390705	2.19521767
HEY2	2.19122286
FLNC	2.1861167
PCDHGA3	2.18359149
SLC22A5	2.18204537
IDUA	2.17912135
LRIG3	2.17777961
TMEM209	2.17727655
CALD1	2.17674845
TMEM170A	2.16863682
PLXNB2	2.16759354
TJP2	2.16451862
SEMA5B	2.15992273
NPC2	2.15858509
DDR2	2.15584501
DDX31	2.15330098
CNR1	2.14934286
PI16	2.14659334
WWTR1	2.14528005
PDE3A	2.14492851
DNAH12	2.14366167
EDNRB	2.13746868
TIMP2	2.13712185
ARHGAP18	2.13403841
KRTCAP3	2.12933932
SLC9A9	2.12906664
CYP7B1	2.11997683
SF3B4	2.11881495
PAFAH1B3	2.11322086
IFI44	2.1127917
LRP2	2.10924392
BTNL9	2.10829621

TXNRD1	2.1066461
RELL1	2.10182283
COL7A1	2.10137269
VWCE	2.09810301
HIST1H2AG	2.0974765
GRP	2.09141679
SLC13A5	2.0908053
DYNLT1	2.08316053
ERAP1	2.07992778
CARD6	2.07670641
AP1G2	2.07209082
CC2D2A	2.06902449
FHL5	2.06889578
THSD4	2.06790396
CASC2	2.06476458
UGT8	2.0641354
RERG	2.06321093
INTU	2.06247488
C12orf60	2.06242305
FMO5	2.06183366
PRR14	2.05884491
CHRAC1	2.05882268
HSD17B3	2.05582558
MCHR1	2.05505344
MICALL2	2.05201546
LRRC1	2.04718923
TP53RK	2.04530515
JAG1	2.04522006
PTGDS	2.04344313
PEX12	2.04196138
C10orf105	2.03881108
TTPA	2.03746752
ARHGAP10	2.02901491
TRIM45	2.01700202
STEAP3	2.01601331
CLTCL1	2.01530211
CARD8	2.00961402
ACACB	2.00437784
SLC27A6	2.00300274
TRPS1	2.00095766
LRFN2	1.99605784
TBX2	1.99563218
PLVAP	1.99520238

COL18A1	1.99416087
FOXC1	1.99229339
IQCD	1.99143674
SLC39A12	1.98912489
ABCA1	1.9861727
BNIP2	1.98373186
ELOVL5	1.98346462
APH1B	1.9828066
MYOM1	1.9822725
TMPRSS5	1.9820127
ISG20	1.97797471
ELL3	1.9740502
CXADRP3	1.97402146
ZNF835	1.97095604
TIMP1	1.97080201
ITGA1	1.97014826
GPR62	1.96801127
HIST1H2AH	1.96724659
KCNJ6	1.96555557
LOC400684	1.96156366
LDB3	1.9559535
PARD3B	1.95267916
ALCAM	1.9488516
H2AFJ	1.94704726
TMEM38B	1.94179798
PLD5	1.94068108
SEC61A1	1.93438392
PCDH18	1.92941392
TAS2R30	1.92652222
PLEKHG2	1.92415672
GPR83	1.92349695
SMURF2	1.92310378
C8orf58	1.92273459
PLAC8L1	1.92184232
TAGLN2	1.92093066
IGF2BP3	1.91558533
STT3A	1.91469413
FAM111A	1.9141154
ANG	1.91145999
DLL1	1.90638782
FKBP5	1.90629177
FREM1	1.90543697
PPP2R3B	1.89999382

CHRNA3	1.89812313
PLTP	1.89612465
ZCCHC3	1.89501787
POLH	1.89466954
CHST14	1.89457693
MORC4	1.89393999
ZC3H4	1.89150249
COL4A2	1.89144479
PLEKHH2	1.88810393
FN1	1.88779562
HEG1	1.88484207
TRRAP	1.88458581
CFD	1.88218566
ADCY4	1.8815612
MALL	1.88106371
PLEKHA7	1.87940241
SEMA3C	1.87816744
S100A6	1.87250317
XKR8	1.87023664
LRIG1	1.86803956
HIST1H2AD	1.86303758
ACCS	1.86301292
DYRK3	1.8614566
HIST1H2AK	1.86116118
TTLL4	1.85678737
PALMD	1.85581896
FOXF2	1.8547792
NPFF	1.85076952
FADS1	1.85067393
C11orf71	1.84756118
MST1	1.84545912
CD163	1.84177618
DSCAML1	1.84142531
GEM	1.83959436
PLP2	1.83959104
LOXL3	1.83854463
HHIP	1.83767201
TJAP1	1.83729437
SMAD5	1.83660396
C1orf56	1.8345601
ZCCHC12	1.83441622
EML3	1.83322012
FUK	1.8298916

PLSCR4	1.82915112
ISOC1	1.8263964
KDM5B	1.82138127
EHD2	1.82110379
LIFR	1.81876383

Table 4 Top 10% genes negatively associated with cortical mean diffusivity

Gene	T
ZBBX	-5.8655597
COL10A1	-4.9772037
RPH3A	-4.7870584
LRRC20	-4.7790418
GALNTL5	-4.7187389
SERPINF1	-4.6996848
PDK3	-4.6960537
KCNIP3	-4.5922382
RP9P	-4.4839389
PHLDA2	-4.398832
BEND5	-4.382623
CDT1	-4.2447609
TM6SF1	-4.1233527
LPL	-4.1044895
NOS2	-4.0990248
PINK1	-4.0962372
KRTAP19-8	-4.0900589
XKR9	-4.0876736
SPHK2	-4.0811417
NEFL	-4.0787576
LYRM4	-4.0755687
FAM162B	-4.0746256
SLAMF7	-4.0468249
KIF17	-4.0418209
COPS7A	-4.0396666
MAPK10	-3.9996099
DCAF8L2	-3.9879531
GHITM	-3.9830406
DUSP3	-3.9422763
KLHDC3	-3.9289819
IPCEF1	-3.9253214
CD79A	-3.8688458
CALM2	-3.8596883
MGLL	-3.8583534
AMN1	-3.8511895
CRYBB2P1	-3.8455618
ALX3	-3.8407556
C3orf18	-3.839471
STRBP	-3.8372414
OR5L2	-3.8306
GNG3	-3.8194455

RHEB	-3.8166614
HOXB13	-3.8090339
CDH8	-3.8077859
HLA-DRB3	-3.7947233
UBE2QL1	-3.7939761
FDX1L	-3.7763963
HAMP	-3.7709883
RIMS1	-3.7429875
SURF2	-3.7321876
LRRK1	-3.727944
ANKRD42	-3.7248841
HRASLS	-3.7232395
BCORP1	-3.7226912
SLC22A7	-3.713603
NRN1	-3.7001958
C3orf14	-3.6949514
IRX5	-3.6865178
ATXN7L3	-3.6786286
DHRS7B	-3.6774447
FRAT2	-3.6730785
PLXDC1	-3.6727155
UBE2D4	-3.6683898
BID	-3.6628081
SLC2A5	-3.6580734
GPATCH4	-3.6564407
ATF4	-3.6557671
TUSC2	-3.6550299
TREM2	-3.6544525
OCIAD2	-3.6427161
DNAJC19	-3.6274626
DBNDD1	-3.6240873
C14orf177	-3.6071964
SPEF1	-3.5990108
COL24A1	-3.5910885
SNRNP25	-3.5883887
CLEC4M	-3.5845657
SPATS2	-3.5835463
STAMBPL1	-3.5794437
CLEC2L	-3.5703084
SAMD14	-3.5703021
TAGLN3	-3.5676901
OPA3	-3.5650386
UCHL3	-3.5625757

SMPX	-3.561534
MED10	-3.5610282
ABCC12	-3.5593704
TMEM232	-3.554067
NCKAP1	-3.5490832
ST8SIA5	-3.5477037
YIPF1	-3.5360179
GPR179	-3.5350107
UROS	-3.5298639
ZNF385B	-3.5296996
CAPS2	-3.5277346
DTX4	-3.522191
PSMG3	-3.5219828
RB1CC1	-3.5196222
PPP1R11	-3.5173623
POP4	-3.5121318
PPM1H	-3.5120143
OAT	-3.5094982
WNT10A	-3.5076684
POPDC3	-3.5045966
UST	-3.5000296
ITGB1BP1	-3.4991998
MYBPHL	-3.4930255
HLA-DRA	-3.4922798
C1orf216	-3.4881014
UGP2	-3.4865481
FBXO9	-3.4828873
SNX32	-3.4816723
IGFBP6	-3.4762212
NARS	-3.4748032
GLMN	-3.4733283
SDCCAG8	-3.4728704
GMPR2	-3.4630842
MICAL2	-3.4614736
C6orf106	-3.4591499
CA10	-3.4551213
RANBP9	-3.4507457
THOC7	-3.4460957
LOC100126784	-3.4457085
ALOX5	-3.4444367
RNF168	-3.4432099
COL5A2	-3.4426424

TIMM17A	-3.4392889
LRRC6	-3.438557
TRNP1	-3.4375234
C3AR1	-3.4325996
PDCL3	-3.4299873
ZNF720	-3.4275251
VTI1B	-3.4213828
STAU2	-3.4201617
HMGCS2	-3.4196324
ATP6V1E2	-3.4123917
SYCP2L	-3.4074836
KAZALD1	-3.4070478
NDUFB9	-3.4059315
RNF214	-3.4001738
CYP2E1	-3.3994706
SAE1	-3.3939098
TOMM5	-3.3810352
RASL10B	-3.3770124
FAM182B	-3.3763827
HSD11B1L	-3.3713955
RPA4	-3.3695793
POLE4	-3.3623392
ARMCX3	-3.3616474
ITPK1	-3.35991
SLC35C1	-3.3544148
SHC3	-3.3534697
CTSS	-3.3506701
TTR	-3.3466778
NHLRC4	-3.3435683
PSMC3	-3.3401919
LRRC39	-3.3340243
POLD4	-3.3293712
TOX	-3.3291598
SH2D2A	-3.3230876
SNN	-3.3217836
ST6GAL1	-3.3216006
KIAA1549	-3.3182591
HLA-DMB	-3.3172422
FNDC4	-3.3134679
TRIM15	-3.3080936
PPP1R14C	-3.3062254
SLC6A12	-3.299078
COX7A2	-3.2915336

NEFM	-3.2909491
BECN1	-3.2885907
KCNQ5	-3.2881012
BCL2L2	-3.2872428
RPS19BP1	-3.2864578
OLFM1	-3.2836959
ZNF143	-3.2812519
COL12A1	-3.2799833
PARD6A	-3.2740508
CIAPIN1	-3.274019
PLEKHM2	-3.2738832
ITGB2	-3.2730424
RGS4	-3.271795
NEFH	-3.271033
HEXIM1	-3.2699336
ADRA1D	-3.2669017
VPS41	-3.2667559
POP7	-3.2646088
CARS	-3.2641428
CCM2	-3.2587769
KIF5A	-3.2553056
ITGB3BP	-3.2523105
SNTG1	-3.2500039
ECT2	-3.2472209
CACNG6	-3.2471107
KCNQ3	-3.2470006
QPCT	-3.2437864
UNC13A	-3.2389445
GLRX5	-3.2371396
ZNF814	-3.2364872
CCDC25	-3.2308117
GPR158	-3.2248055
COQ10B	-3.2224284
BOLA3	-3.221223
AMACR	-3.2183421
PHYH	-3.2162932
RIMKLA	-3.2160119
PITRM1	-3.2133559
AVEN	-3.2052583
ZBTB8OS	-3.2024552
LCE3C	-3.2004237
UBL4A	-3.1976092
ZSWIM7	-3.1961201

ZNF622	-3.1939562
NOMO1	-3.193099
PEF1	-3.1921605
PIN1	-3.1899783
CCNDBP1	-3.1875522
CNTNAP2	-3.1865165
NUDT15	-3.1862165
PTPRT	-3.1822965
ZBTB22	-3.1809087
DENR	-3.1786046
TNFAIP1	-3.1780485
PMPCB	-3.1765259
PAK1	-3.1745147
LSM10	-3.1742813
WDR37	-3.1704636
C12orf10	-3.1660301
SLC25A5	-3.1648261
INSM2	-3.1645503
TPI1	-3.164391
NDUFB8	-3.1602704
LINC00319	-3.1594763
METTL13	-3.1583513
NAALADL2	-3.1549221
NDUFB3	-3.1548602
RAB3D	-3.1529541
PDXP	-3.1529504
NELL1	-3.1513976
TMSB4X	-3.1512123
IMMT	-3.1494664
TIMM23	-3.1485231
PAAF1	-3.1448611
FAM43B	-3.1403015
ZNF25	-3.1397585
MRPS15	-3.1386009
MANBAL	-3.1354403
CEND1	-3.1329066
CDCA8	-3.1327119
C12orf49	-3.1323094
COX7A1	-3.1306386
CGREF1	-3.1302808
HOXD9	-3.126221
MAT2B	-3.1258023
EPHB6	-3.1229899

PTEN	-3.120597
NPM2	-3.1153899
GNB5	-3.1153318
XK	-3.1140426
TRUB1	-3.1084473
RAD1	-3.1075581
ADORA1	-3.1074817
MRPL11	-3.1070203
KCNJ12	-3.1044501
MAGEA6	-3.103821
ODC1	-3.1031378
C2	-3.1004445
CHCHD4	-3.0990809
UCHL5	-3.0990026
HABP4	-3.0978301
MIR22HG	-3.0965323
PTPN22	-3.0958233
HAGH	-3.0949602
AGMAT	-3.0942233
ANKRD33B	-3.0939413
HYLS1	-3.092776
TOMM22	-3.0922473
VPS26A	-3.0914358
TIMM8A	-3.0864952
BCAP31	-3.0856896
ST6GALNAC6	-3.0803902
C16orf95	-3.080255
SDHB	-3.0781025
CYCS	-3.0736571
ISPD	-3.0691856
ARHGAP25	-3.063869
IMP4	-3.0587017
FAM89B	-3.0584486
HARS	-3.0571914
BMS1	-3.0569092
RPL7	-3.0555406
CLN6	-3.0528616
TRIM41	-3.0498377
DMXL2	-3.0490833
FBXL16	-3.0477567
SPIB	-3.0450003
GGN	-3.0437146

MAP1B	-3.0433452
CMC1	-3.0419273
SCN8A	-3.04158
SERPINB2	-3.0415013
SEC24D	-3.0414304
RABEPK	-3.0396739
TAF13	-3.0367734
MFSD11	-3.0362661
FARSA	-3.0338896
UQCRH	-3.0331346
GLO1	-3.032069
TMEM38A	-3.029478
FLRT3	-3.0277187
RUNDC3A	-3.0269121
LCE1C	-3.0227132
TATDN2	-3.0218022
OTUD6A	-3.0214787
FAM32A	-3.0205459
RDH12	-3.0190742
SAP30L	-3.0174301
AAK1	-3.0173688
C11orf74	-3.0168268
TATDN1	-3.0152343
TMEM132C	-3.0144846
SLC39A13	-3.0143978
DPT	-3.0108119
ANKRD34A	-3.0107554
LYRM7	-3.0100737
ADO	-3.0099907
PFDN4	-3.0098491
VPS37A	-3.0097798
HIGD1B	-3.0070244
KCNV1	-3.0054493
DYTN	-3.0053672
OR2W5	-3.0034317
CCKBR	-3.0013705
CHAF1A	-3.0003092
SLC22A9	-2.9999556
FOXRED1	-2.9988433
CDKL3	-2.9987014
CISD1	-2.9977168
SUSD5	-2.9972606
CPLX1	-2.9972079

ESM1	-2.9969919
UPK3B	-2.9968922
DLD	-2.9968551
MRPL53	-2.9968073
AARS	-2.9957761
MPG	-2.9952696
MRPL15	-2.9923019
ARHGAP26	-2.9914209
NSMCE2	-2.9890005
RAB15	-2.9846225
OSTF1	-2.9796759
WDYHV1	-2.9791214
CBX7	-2.9775215
FMR1	-2.9769321
PPP3R1	-2.9754366
CIDECP	-2.9753592
PGP	-2.9736037
B3GNT6	-2.97101
UBLCP1	-2.9706327
NDUFA12	-2.9692105
NAP1L5	-2.9674989
PRUNE2	-2.9670344
PTPN4	-2.9664737
RPL37A	-2.9629367
DCK	-2.9614573
RNF157	-2.9598371
GPR108	-2.9598209
LAMB3	-2.9593732
VDAC2	-2.9577019
CCDC3	-2.9576254
FBXO45	-2.9569135
PCSK1	-2.9565875
LETMD1	-2.9565396
ING1	-2.9564847
TMX4	-2.9548158
SUPT3H	-2.9540816
SNX17	-2.9538807
RFTN1	-2.9532042
COX4I1	-2.9526547
GRK4	-2.9513881
DEF8	-2.9507727
CFL1P1	-2.9501678
DENND4B	-2.9493077

CA13	-2.9482297
ISX	-2.9473088
TRAK2	-2.9472445
ETS2	-2.9469902
NCALD	-2.9462042
ITPA	-2.9440395
KHDC1	-2.9417446
ZNF576	-2.9410736
FRS3	-2.9399925
AQP7P1	-2.9396032
OXR1	-2.9384721
RASGRP2	-2.9370921
TRMT61B	-2.933693
USE1	-2.9327809
STOML2	-2.9323424
SNRPA1	-2.9304029
DNAJA2	-2.9298551
PRMT5	-2.9263542
CTNNA2	-2.9262565
PCDHB15	-2.9250645
F8	-2.9234077
RPUSD4	-2.9214044
HSPA9	-2.9208829
SMARCC2	-2.9188935
UQCRFS1	-2.9188521
TMX3	-2.9187129
OSBPL1A	-2.9181809
COX5A	-2.914463
AIF1	-2.9142186
ZNF593	-2.9136821
SLC25A26	-2.9115827
SLC38A6	-2.9109502
GAS5	-2.9096722
NARF	-2.9089825
BMP10	-2.90601
RPL9	-2.9059926
MCAT	-2.9045671
HIGD1A	-2.9010368
PPP1R12A	-2.8996641
EIF2B5	-2.8982553
GABARAPL3	-2.8952401
SLC25A3	-2.893825

ABCF1	-2.8923317
RGS7	-2.8922156
MRPS22	-2.892172
TCTE1	-2.8917361
TUBG2	-2.8914627
ASGR2	-2.8899517
KCNMB4	-2.8892843
FCER1G	-2.8886771
ATP6V1G2	-2.8872671
KRT222	-2.8867537
STK39	-2.8831464
NEDD8	-2.8825156
SNRNP27	-2.8823623
GOT1	-2.8822738
TPMT	-2.8812801
CCT6P1	-2.8809917
PSMD12	-2.8808875
NGLY1	-2.8790277
TAF9	-2.878323
FAM168B	-2.87804
FAM20A	-2.8775358
ACTR1B	-2.876342
RIT2	-2.8756852
KLHL8	-2.8755231
RHOBTB2	-2.8753661
C7orf57	-2.8737425
CCDC43	-2.8719389
PFKM	-2.8704867
TUBG1	-2.8692839
MTX2	-2.8682065
RSRC1	-2.8681108
UQCRHL	-2.8669789
ENTPD4	-2.8665177
HECA	-2.8649216
RPL29P2	-2.8649079
ZNF223	-2.8632976
BBS7	-2.862998
POLDIP2	-2.862425
MIR205HG	-2.8618493
TXNL4A	-2.8616084
DCTN6	-2.8601309
CHI3L1	-2.8591961
ZNF213	-2.8546363

KLK9	-2.8532133
CRYM	-2.8513979
SULT2B1	-2.8501127
ZNF517	-2.8497915
MFF	-2.8486746
RNFT2	-2.8485507
SGTB	-2.8484973
DENND2D	-2.8455464
DRG1	-2.8450514
TRIM44	-2.8450299
PDPN	-2.8434371
TPK1	-2.8417366
STAT6	-2.8406249
LDB2	-2.8404259
ENDOG	-2.8395742
SERPINI1	-2.838105
RP9	-2.8337881
ESRRA	-2.8320352
HNF1A-AS1	-2.8319377
ST8SIA1	-2.8316774
DGKI	-2.8287841
FARS2	-2.8278116
ZFAND2A	-2.8258835
RCHY1	-2.8248237
IRAK1	-2.824327
MPPED1	-2.8242948
NME5	-2.8229657
AQP11	-2.8223731
ERCC1	-2.8213623
ZCRB1	-2.8200021
ZW10	-2.8197166
RNASEH2B	-2.8178685
PGAM1	-2.8178172
SLC4A9	-2.817338
DHRS11	-2.8158844
SERGEF	-2.8151598
KCNH1	-2.8150025
TOMM20L	-2.814146
AKTIP	-2.8138121
BNIP3	-2.8113379
KLK14	-2.8111149
MGST3	-2.8100351
DRAP1	-2.8076676

ATP1B3	-2.8050192
TERF2	-2.8048712
SATB1	-2.8040941
RHO	-2.8039427
HLA-DRB1	-2.8027065
WSB2	-2.8018908
CA11	-2.801106
RGS10	-2.8009734
TMEM163	-2.7992869
RPL31P11	-2.7986664
PPP3R2	-2.7977942
CDC123	-2.7970828
NMNAT2	-2.7961198
DYNC1I1	-2.7960227
MRPS18C	-2.7955366
FAM3C	-2.7955125
TM2D1	-2.7942331
FKBP3	-2.7920511
NDUFS5	-2.791734
SH3KBP1	-2.7896461
SCYL1	-2.7888954
OXA1L	-2.7877594
TM7SF3	-2.7854621
AHNAK2	-2.7850014
A2ML1	-2.7838576
ASB13	-2.7830114
CAPZA2	-2.7828072
ARSG	-2.782224
ABI3	-2.7819786
FAP	-2.7816527
GSTZ1	-2.7813813
CHN1	-2.7808479
EML6	-2.7807637
HLA-DPA1	-2.7802845
SPSB4	-2.7801353
PSMC5	-2.7773812
TRUB2	-2.7764754
MPND	-2.7752362
ARL6IP4	-2.7751111
PRPSAP1	-2.7750009
RPP40	-2.7747521
LARP1B	-2.7743731
MYO5A	-2.7722897

POLR2E	-2.7716118
HCRTR1	-2.7710502
B4GALT6	-2.7700447
STXBP5L	-2.7683589
FBXO6	-2.7683296
AGBL2	-2.7671152
FLVCR1-AS1	-2.7661802
PACRG	-2.7660257
DDHD2	-2.7655324
TMEM150B	-2.7645188
CETN4P	-2.7645008
TUBA8	-2.7631009
FKBP1B	-2.7627268
C21orf58	-2.761731
NIPSNAP1	-2.7614464
GPR78	-2.7607427
TLN2	-2.7604562
ALPP	-2.7590278
GRIA3	-2.7581289
FGF12	-2.757237
DPP10	-2.7571413
ZIC4	-2.7551814
UMOD	-2.7550965
GRK5	-2.7523248
CARTPT	-2.7513172
TFPT	-2.7504056
EXTL1	-2.7488631
KIF3B	-2.7488555
WASF3	-2.7487558
PPP2R2C	-2.7487013
GPR153	-2.7485733
OPTN	-2.7483694
GGNBP2	-2.7468265
COG6	-2.7467625
MECR	-2.7460891
ANKRD52	-2.7436556
UBE2F	-2.7432943
ZNF358	-2.7427356
HAR1A	-2.7426083
KATNB1	-2.7396266
RAP1GDS1	-2.7389063
CTSD	-2.7388537

ERN2	-2.738271
PTRHD1	-2.7382219
PSMD6	-2.7378406
PPP1CA	-2.737131
PLEKHA8	-2.7362217
NTN4	-2.7357592
ZBTB26	-2.7331454
OLFM3	-2.7331015
SLC35F3	-2.7324128
TSR1	-2.7313001
PRDM10	-2.7306943
GMFB	-2.7293993
TMEM126A	-2.726737
PIP4K2C	-2.7266375
VSNL1	-2.7261276
CDH22	-2.7234324
POLR1E	-2.7223714
MLST8	-2.7197907
NNT	-2.7186096
LY6E	-2.7185202
PYY2	-2.7182252
DDX1	-2.7179384
RRP12	-2.7172949
PPM1A	-2.7158605
PLCL2	-2.7155626
COASY	-2.7149953
SYTL2	-2.7143944
SV2C	-2.711352
SCGB1A1	-2.7102053
LOC407835	-2.7097351
EIF4G1	-2.7095064
YWHAG	-2.7088348
CAPNS1	-2.7085817
RND2	-2.7084827
KIF20A	-2.7077901
ACTN2	-2.7075992
MTIF2	-2.7065182
TMEM155	-2.7050295
BUD31	-2.7049185
CHMP5	-2.7042815
SERP2	-2.7037803
BAP1	-2.7022215
CCNI	-2.700584

CCDC39	-2.6991178
CYTH4	-2.6985828
HDDC2	-2.6985553
MRPL37	-2.6979951
ZDHHC17	-2.6976756
ROPN1L	-2.6963491
NDUFA4	-2.6961294
MRTO4	-2.6955612
ERRFI1	-2.6952509
AZI2	-2.6936839
COL19A1	-2.6907982
PEX11B	-2.6885281
KCTD9	-2.6847387
SLC17A9	-2.6842744
SLAMF8	-2.6833399
CORO2A	-2.6830872
RTKN2	-2.6828184
FITM1	-2.6817405
COPS8	-2.6810386
VAPB	-2.6808581
CBX6	-2.6806602
FAM49B	-2.6801498
RPL21P44	-2.6793414
YARS	-2.6781928
CD53	-2.6780712
NT5M	-2.6775535
NAP1L2	-2.6771026
ZEB1	-2.6766573
SDHD	-2.6762086
CDK5	-2.6755352
MAD2L1BP	-2.675333
RTN4RL2	-2.6745079
ATP6V0E2	-2.6731728
KNG1	-2.6726878
KCNA2	-2.6724103
VAX2	-2.6723635
PPP1R15B	-2.672055
RTN2	-2.6716894
RHCE	-2.671177
FAM57B	-2.6707769
DLGAP1	-2.6706033
SUCLA2	-2.6699873
UQCRC1	-2.6695442

MTFMT	-2.6689673
SORCS1	-2.668203
FBXL15	-2.6670353
TDRD7	-2.6663523
ZNHIT3	-2.666337
TUBB8	-2.6646364
PCMT1	-2.6646127
RASD1	-2.664271
CASQ1	-2.6639485
MTRF1L	-2.6628615
CHML	-2.6626317
ICA1	-2.6621383
TMEM173	-2.6614419
MAN2B2	-2.6609641
CSF3R	-2.6597524
SERINC3	-2.6593319
TSPAN7	-2.6590708
CCL25	-2.6573376
ALKBH6	-2.6570435
NDUFB5	-2.6569323
PCGF3	-2.6553856
MCOLN1	-2.6544028
CPLX2	-2.6529525
FEM1A	-2.6522167
TYROBP	-2.6521717
ASB4	-2.6503084
TMEM191A	-2.6500164
DALRD3	-2.6497111
GAP43	-2.6493235
MPP6	-2.6460541
PITPNM1	-2.6459434
HINT1	-2.6453678
CCDC124	-2.6444246
NGEF	-2.644377
AKT3	-2.6440423
NRIP3	-2.6438811
WDR45	-2.64139
RSPH3	-2.6396979
IDH3B	-2.6385656
ELAVL4	-2.6383551
APEH	-2.636391
TRAF3IP3	-2.6362758
PRKAB1	-2.6349174

NDUFV2	-2.6347833
CCND2	-2.6328885
CHMP4A	-2.6323448
VAMP1	-2.6322344
PPP2R2D	-2.6319321
RNASEH1	-2.6317499
MRPL12	-2.6313425
C12orf43	-2.6295372
OR4K2	-2.6284741
NDUFA7	-2.6267802
PELI3	-2.6259826
ME3	-2.6259797
RPSAP52	-2.6259369
RPF2	-2.6252764
TWF2	-2.6242597
UBE2E2	-2.623375
IL17RE	-2.6224403
DCTD	-2.6215237
ARHGEF4	-2.6201438
MMADHC	-2.6196376
ST13P4	-2.619548
WDR93	-2.6190425
ASS1	-2.618842
TBC1D9	-2.618477
FEZ2	-2.6184573
TASP1	-2.6179414
CALM3	-2.6178629
C9orf163	-2.6177007
NAT8L	-2.6173793
XCR1	-2.6157607
STX1A	-2.6156634
NPW	-2.6156591
TOLLIP	-2.6154364
MAP3K10	-2.6147907
SLC25A11	-2.6146234
TUBA1A	-2.6146003
TCTEX1D1	-2.6140032
CCNB1	-2.6139739
COL11A1	-2.6134878
COMMD9	-2.6132491
RWDD2B	-2.6119905
SYNGR3	-2.6113128
GPX5	-2.6110185

KCNJ8	-2.610291
SBDSP1	-2.6098307
C1orf123	-2.6092331
MNAT1	-2.6084234
LSM1	-2.6083064
MTCH2	-2.6079945
FBXW5	-2.6079365
FAM9B	-2.6079124
GLRX	-2.6072725
CXCL3	-2.606671
GLRX3	-2.6062488
ARMC6	-2.6060692
FBXO16	-2.6036095
IDNK	-2.602198
RAD23B	-2.6015554
HMGB3	-2.6014787
HSPB3	-2.5985089
BAG1	-2.5975532
DPP8	-2.597103
RNF14	-2.5962628
MRPL51	-2.5961981
C9orf129	-2.5959534
DNAJC4	-2.592662
GPR161	-2.5917895
GUK1	-2.5908548
TAF1D	-2.5905283
TMEM8B	-2.5904412
CISD3	-2.5896221
CENPK	-2.5893982
SLC30A9	-2.5888733
NCK2	-2.5886757
PPP1R3F	-2.5875002
PAIP2	-2.5858666
SPDEF	-2.5840326
RPS27	-2.583432
MTUS2	-2.5820604
NSDHL	-2.5817982
DYNLT3	-2.5817038
MRPL48	-2.5806448
GOLGA5	-2.5792632
FAM135B	-2.579032
EMC3	-2.5788407
ENPP5	-2.5784064

ITM2B	-2.578375
NDUFAF1	-2.5771953
PKD2L1	-2.5763228
ETV6	-2.575834
APOL2	-2.575735
RNF216	-2.5747205
CNIH3	-2.5729808
UBAC2	-2.5728112
DUS1L	-2.5718428
DND1	-2.5709478
NOA1	-2.570822
ARMC10	-2.5706624
NIPA1	-2.5697766
SARS	-2.5684993
PRDM11	-2.5684252
TTTY15	-2.5682419
RPUSD1	-2.5679161
CALML3	-2.5661886
NPHS2	-2.5649582
CDC26	-2.5647044
CCDC91	-2.5646253
ZC4H2	-2.5645834
PFN1	-2.5640967
PPP2CB	-2.5632099
ACHE	-2.562719
LMO1	-2.562321
TMEM141	-2.5617051
NAPSB	-2.5586997
ABTB1	-2.558554
STEAP2	-2.5571628
OCEL1	-2.5570403
PSMC2	-2.5567874
MYBBP1A	-2.5557077
OR2W3	-2.5547983
EIF3A	-2.5546006
TTC39A	-2.5543505
KRR1	-2.5534962
TOMM40L	-2.5527767
PGAM1P5	-2.5526289
LALBA	-2.5525236
NDUFB2	-2.5520427
TMEM14B	-2.5501817
SYNJ1	-2.5493331

C2orf69	-2.5482566
MRPS28	-2.5480501
NANOS3	-2.5473365
SMS	-2.5471948
TAF4B	-2.5468145
SHD	-2.5466571
CHAC1	-2.546512
SNX3	-2.5446162
SLC4A1AP	-2.5435582
VPS13A	-2.5426343
MYEF2	-2.5413768
CYP1B1-AS1	-2.5412827
CDR2	-2.5405092
ENTPD6	-2.5395428
RAB42	-2.5390372
AKAP12	-2.5380503
OPN4	-2.5376786
ZFP91	-2.5373217
PPIL3	-2.5365304
AQP2	-2.536469
OR10H5	-2.5364018
WDR41	-2.5363866
DVL1	-2.5362119
SPRR2E	-2.535224
DCTN1	-2.5341129
NLRC5	-2.5341045
EPHA3	-2.5338038
FBXW7	-2.5334906
GNA15	-2.5324698
RARB	-2.531893
SLC25A46	-2.5317661
FIBP	-2.5317024
C8orf33	-2.5312157
C1orf158	-2.5304941
MALSU1	-2.5299868
APOOL	-2.5297531
ATCAY	-2.5284561
GDF7	-2.5276145
OR4D10	-2.5272106
TRMT12	-2.5267569
COQ10A	-2.5263452
CHMP4C	-2.526281

LRRC15	-2.5259993
C1QB	-2.5245338
MTO1	-2.5241246
PCDHAC2	-2.5236821
MDH2	-2.5228979
ZNF582	-2.5221604
SLC6A13	-2.5216393
NKX2-3	-2.5208598
RALBP1	-2.5201691
GGCT	-2.5191475
HMG20A	-2.5191016
ACTR6	-2.5167339
ARPC5L	-2.5161856
SEH1L	-2.515748
NDUFS6	-2.5150187
CTNNBIP1	-2.5146357
MCF2L2	-2.5130667
CACNB1	-2.5125411
NDUFA6	-2.5122025
TAF6	-2.512187
CIDEC	-2.5120141
OXNAD1	-2.5115627
ZSCAN5A	-2.5114533
STMN2	-2.510632
HPRT1	-2.5097577
H1FNT	-2.5083273
NDUFB4	-2.5071608
IMP3	-2.5070751
MKKS	-2.5066575
THUMPD2	-2.5065912
MEX3D	-2.5061434
CTSK	-2.5043467
HCRT	-2.5043342
GDF10	-2.5040214
PLA2G5	-2.5038494
DOPEY2	-2.5035742
KCNAB1	-2.50219
RAPGEF1	-2.5011066
GRPEL2	-2.4997267
LY9	-2.4992676
BRK1	-2.4988563
ATG16L2	-2.4984735
SCN2B	-2.4976452

PTP4A1	-2.4975567
SLAMF9	-2.4957508
DYDC2	-2.4927855
GNB1	-2.4922636
CAMK4	-2.4919338
TRAF3IP1	-2.4914162
PIH1D1	-2.4912981
EEF1B2	-2.4909193
ALOX5AP	-2.4897621
SYAP1	-2.4890887
EIF4E1B	-2.4888188
SUSD3	-2.4870549
FBXW2	-2.4858543
DNAJC18	-2.4846971
PI4KA	-2.4838714
ELP3	-2.4837308
EN2	-2.4835492
SENP2	-2.4833341
LDHA	-2.4829614
HNRNPD	-2.4828908
OR5V1	-2.4825414
PLA2G7	-2.4822932
UBE3A	-2.4822572
GBA2	-2.4817666
RAB4A	-2.4806329
APBB1IP	-2.4797377
FSTL4	-2.4795916
OR2H1	-2.4793292
PREP	-2.4784388
GLRB	-2.4784239
MRPS14	-2.4779189
AGTPBP1	-2.4773223

Table 5 GO terms enriched in the top 10% of genes positively associated with cortical volume in preHD

GO Term	Description	P value
GO:0032501	multicellular organismal process	1.21E-27
GO:0048513	animal organ development	2.17E-27
GO:0048856	anatomical structure development	2.07E-26
GO:0007275	multicellular organism development	5.51E-26
GO:0009653	anatomical structure morphogenesis	2.83E-25
GO:0032502	developmental process	3.87E-25
GO:0048731	system development	1.24E-24
GO:0009888	tissue development	1.05E-23
GO:0009887	animal organ morphogenesis	4.94E-23
GO:0072359	circulatory system development	7.56E-20
GO:0050896	response to stimulus	1.76E-18
GO:0065007	biological regulation	2.52E-16
GO:0048646	anatomical structure formation involved in morphogenesis	3.84E-16
GO:0060429	epithelium development	1.55E-15
GO:0048869	cellular developmental process	2.14E-15
GO:0030154	cell differentiation	2.47E-15
GO:0007154	cell communication	6.77E-15
GO:0050794	regulation of cellular process	8.51E-15
GO:0023052	signaling	1.69E-14
GO:0035295	tube development	1.92E-14
GO:0051716	cellular response to stimulus	1.28E-13
GO:0007165	signal transduction	9.66E-13
GO:0050789	regulation of biological process	1.56E-12
GO:0030198	extracellular matrix organization	2.50E-12
GO:0043062	extracellular structure organization	2.78E-12
GO:0045229	external encapsulating structure organization	3.44E-12
GO:0001944	vasculature development	3.96E-12
GO:0035239	tube morphogenesis	4.24E-12
GO:0016477	cell migration	5.54E-12
GO:0001568	blood vessel development	6.94E-12
GO:0050793	regulation of developmental process	7.59E-12
GO:0009790	embryo development	1.20E-11
GO:0008283	cell population proliferation	1.83E-11
GO:0030855	epithelial cell differentiation	4.68E-11
GO:0042127	regulation of cell population proliferation	1.19E-10
GO:0007507	heart development	1.91E-10
GO:0007166	cell surface receptor signaling pathway	2.50E-10
GO:0061061	muscle structure development	3.28E-10
GO:0051239	regulation of multicellular organismal process	4.46E-10
GO:0048514	blood vessel morphogenesis	7.02E-10

GO:0048518	positive regulation of biological process	9.37E-10
GO:0048729	tissue morphogenesis	1.10E-09
GO:0042221	response to chemical	1.59E-09
GO:0051674	localization of cell	1.67E-09
GO:0048870	cell motility	1.67E-09
GO:0001655	urogenital system development	3.25E-09
GO:0003007	heart morphogenesis	1.07E-08
GO:0048522	positive regulation of cellular process	1.37E-08
GO:0048523	negative regulation of cellular process	2.06E-08
GO:0003013	circulatory system process	6.99E-08
GO:0007155	cell adhesion	8.15E-08
GO:0065008	regulation of biological quality	8.86E-08
GO:0030334	regulation of cell migration	8.88E-08
GO:0009605	response to external stimulus	1.10E-07
GO:0040011	locomotion	1.14E-07
GO:0003018	vascular process in circulatory system	1.21E-07
GO:0072001	renal system development	1.62E-07
GO:0001822	kidney development	3.31E-07
GO:0040012	regulation of locomotion	4.12E-07
GO:0010646	regulation of cell communication	4.23E-07
GO:2000026	regulation of multicellular organismal development	5.80E-07
GO:0040017	positive regulation of locomotion	6.23E-07
GO:0048583	regulation of response to stimulus	6.80E-07
GO:0048468	cell development	9.71E-07
GO:0003008	system process	1.08E-06
GO:0007399	nervous system development	1.11E-06
GO:0023051	regulation of signaling	1.12E-06
GO:2000145	regulation of cell motility	1.17E-06
GO:0001525	angiogenesis	1.94E-06
GO:0030335	positive regulation of cell migration	2.17E-06
GO:0030199	collagen fibril organization	3.03E-06
GO:0051270	regulation of cellular component movement	3.68E-06
GO:0061448	connective tissue development	4.03E-06
GO:0001503	ossification	4.09E-06
GO:0001501	skeletal system development	4.29E-06
GO:0010033	response to organic substance	5.08E-06
GO:0051272	positive regulation of cellular component movement	5.64E-06
GO:0070887	cellular response to chemical stimulus	6.17E-06
GO:2000147	positive regulation of cell motility	8.27E-06
GO:0032879	regulation of localization	1.08E-05
GO:0010604	positive regulation of macromolecule metabolic process	1.18E-05
GO:0006928	movement of cell or subcellular component	1.37E-05
GO:0048568	embryonic organ development	1.60E-05

GO:0051254	positive regulation of RNA metabolic process	1.85E-05
GO:0009893	positive regulation of metabolic process	2.48E-05
GO:0009966	regulation of signal transduction	2.99E-05
GO:0043588	skin development	2.99E-05
GO:0007204	positive regulation of cytosolic calcium ion concentration	3.07E-05
GO:0002009	morphogenesis of an epithelium	4.13E-05
GO:1902680	positive regulation of RNA biosynthetic process	4.70E-05
GO:0048519	negative regulation of biological process	5.00E-05
GO:0008284	positive regulation of cell population proliferation	5.13E-05
GO:0072132	mesenchyme morphogenesis	5.35E-05
GO:0003170	heart valve development	5.40E-05
GO:0009792	embryo development ending in birth or egg hatching	5.70E-05
GO:0007423	sensory organ development	5.75E-05
GO:0043009	chordate embryonic development	6.11E-05
GO:0048598	embryonic morphogenesis	6.30E-05
GO:0003179	heart valve morphogenesis	6.68E-05
GO:0045944	positive regulation of transcription by RNA polymerase II	6.98E-05
GO:0072006	nephron development	7.48E-05
GO:0045893	positive regulation of transcription, DNA-templated	8.28E-05
GO:1903508	positive regulation of nucleic acid-templated transcription	8.28E-05
GO:0031328	positive regulation of cellular biosynthetic process	8.37E-05
GO:0006950	response to stress	9.69E-05
GO:0009891	positive regulation of biosynthetic process	9.99E-05
GO:0051093	negative regulation of developmental process	0.00011
GO:0051240	positive regulation of multicellular organismal process	0.000112
GO:2001236	regulation of extrinsic apoptotic signaling pathway	0.000129
GO:0045446	endothelial cell differentiation	0.000136
GO:0010647	positive regulation of cell communication	0.000144
GO:0045595	regulation of cell differentiation	0.000148
GO:0042592	homeostatic process	0.000149
GO:0023056	positive regulation of signaling	0.00016
GO:0060537	muscle tissue development	0.000174
GO:0003158	endothelium development	0.000189
GO:0010557	positive regulation of macromolecule biosynthetic process	0.00019
GO:0008015	blood circulation	0.000205
GO:0045935	positive regulation of nucleobase-containing compound metabolic process	0.000219
GO:0072009	nephron epithelium development	0.000259
GO:0050673	epithelial cell proliferation	0.000275
GO:0042692	muscle cell differentiation	0.000287
GO:0034329	cell junction assembly	0.000296
GO:0051094	positive regulation of developmental process	0.000398
GO:0009968	negative regulation of signal transduction	0.000411
GO:0051216	cartilage development	0.000445

GO:0031325	positive regulation of cellular metabolic process	0.000494
GO:0060485	mesenchyme development	0.000568
GO:0051241	negative regulation of multicellular organismal process	0.000613
GO:0001667	ameboidal-type cell migration	0.000665
GO:0048584	positive regulation of response to stimulus	0.000705
GO:0022008	neurogenesis	0.000798
GO:0001570	vasculogenesis	0.000821
GO:0085029	extracellular matrix assembly	0.000997
GO:0048585	negative regulation of response to stimulus	0.001069
GO:0071310	cellular response to organic substance	0.001075
GO:0033002	muscle cell proliferation	0.001162
GO:0072073	kidney epithelium development	0.001204
GO:0071363	cellular response to growth factor stimulus	0.001258
GO:0060840	artery development	0.001459
GO:0006935	chemotaxis	0.001516
GO:0042330	taxis	0.001646
GO:0035850	epithelial cell differentiation involved in kidney development	0.001865
GO:1901888	regulation of cell junction assembly	0.002051
GO:0048771	tissue remodeling	0.002214
GO:0007517	muscle organ development	0.002361
GO:0045216	cell-cell junction organization	0.002594
GO:0050678	regulation of epithelial cell proliferation	0.002646
GO:0009719	response to endogenous stimulus	0.002872
GO:0097191	extrinsic apoptotic signaling pathway	0.002945
GO:0032101	regulation of response to external stimulus	0.003143
GO:0050900	leukocyte migration	0.003153
GO:0080090	regulation of primary metabolic process	0.003378
GO:0022603	regulation of anatomical structure morphogenesis	0.003721
GO:0051173	positive regulation of nitrogen compound metabolic process	0.003722
GO:0035296	regulation of tube diameter	0.003998
GO:0097746	blood vessel diameter maintenance	0.003998
GO:0070848	response to growth factor	0.004006
GO:0040007	growth	0.004156
GO:0007186	G protein-coupled receptor signaling pathway	0.00423
GO:1903596	regulation of gap junction assembly	0.004349
GO:0035150	regulation of tube size	0.004399
GO:0023057	negative regulation of signaling	0.004405
GO:0003206	cardiac chamber morphogenesis	0.004475
GO:0007267	cell-cell signaling	0.00469
GO:0048863	stem cell differentiation	0.005195
GO:0060562	epithelial tube morphogenesis	0.005385
GO:0080134	regulation of response to stress	0.005691
GO:0048738	cardiac muscle tissue development	0.006341

GO:0009967	positive regulation of signal transduction	0.007231
GO:0010648	negative regulation of cell communication	0.007689
GO:0048844	artery morphogenesis	0.008075
GO:0007043	cell-cell junction assembly	0.009161
GO:0065009	regulation of molecular function	0.009873
GO:0048562	embryonic organ morphogenesis	0.010144
GO:0008285	negative regulation of cell population proliferation	0.010926
GO:0031323	regulation of cellular metabolic process	0.010946
GO:0061005	cell differentiation involved in kidney development	0.011095
GO:0030182	neuron differentiation	0.011118
GO:0003197	endocardial cushion development	0.011632
GO:0034330	cell junction organization	0.01192
GO:0002064	epithelial cell development	0.012683
GO:0001935	endothelial cell proliferation	0.01296
GO:0009612	response to mechanical stimulus	0.014595
GO:0048660	regulation of smooth muscle cell proliferation	0.01561
GO:0003171	atrioventricular valve development	0.015818
GO:0051146	striated muscle cell differentiation	0.015887
GO:0014706	striated muscle tissue development	0.01762
GO:0007389	pattern specification process	0.018105
GO:0009611	response to wounding	0.018108
GO:0003012	muscle system process	0.019098
GO:1903598	positive regulation of gap junction assembly	0.019597
GO:0048880	sensory system development	0.020252
GO:0042981	regulation of apoptotic process	0.020331
GO:0003272	endocardial cushion formation	0.020585
GO:0003151	outflow tract morphogenesis	0.020904
GO:0043067	regulation of programmed cell death	0.020927
GO:0042063	gliogenesis	0.021828
GO:2001233	regulation of apoptotic signaling pathway	0.022152
GO:0098609	cell-cell adhesion	0.022765
GO:0048659	smooth muscle cell proliferation	0.024207
GO:0055001	muscle cell development	0.024724
GO:0048589	developmental growth	0.025136
GO:0045597	positive regulation of cell differentiation	0.025823
GO:0003231	cardiac ventricle development	0.026538
GO:0031324	negative regulation of cellular metabolic process	0.027618
GO:0048699	generation of neurons	0.029955
GO:0032835	glomerulus development	0.031272
GO:0060993	kidney morphogenesis	0.033595
GO:0051179	localization	0.034515
GO:0010001	glial cell differentiation	0.034754
GO:0001654	eye development	0.035581

GO:0048608	reproductive structure development	0.036926
GO:2001237	negative regulation of extrinsic apoptotic signaling pathway	0.037381
GO:0009889	regulation of biosynthetic process	0.039225
GO:0006954	inflammatory response	0.040227
GO:0031326	regulation of cellular biosynthetic process	0.041333
GO:0010941	regulation of cell death	0.041803
GO:0061458	reproductive system development	0.041942
GO:0001701	in utero embryonic development	0.042643
GO:0150063	visual system development	0.042643
GO:0051171	regulation of nitrogen compound metabolic process	0.043863
GO:0071495	cellular response to endogenous stimulus	0.045963
GO:0003205	cardiac chamber development	0.046208

Table 6 GO terms enriched in the top 10% of genes negatively associated with cortical volume in preHD

GO Term	Description	P value
GO:0044260	cellular macromolecule metabolic process	2.06E-06
GO:1901564	organonitrogen compound metabolic process	1.38E-05
GO:0019538	protein metabolic process	8.52E-05
GO:0044267	cellular protein metabolic process	9.96E-05
GO:0006793	phosphorus metabolic process	0.00037327
GO:0006796	phosphate-containing compound metabolic process	0.00044584
GO:0043412	macromolecule modification	0.00119709
GO:0099536	synaptic signaling	0.00272483
GO:0006470	protein dephosphorylation	0.00302114
GO:0048167	regulation of synaptic plasticity	0.00565147
GO:0036211	protein modification process	0.01119971
GO:0006464	cellular protein modification process	0.01119971
GO:0007399	nervous system development	0.02198707
GO:0050806	positive regulation of synaptic transmission	0.02356441
GO:0035335	peptidyl-tyrosine dephosphorylation	0.02959106
GO:0007268	chemical synaptic transmission	0.03163031
GO:0098916	anterograde trans-synaptic signaling	0.03163031
GO:0009987	cellular process	0.03712352
GO:0099537	trans-synaptic signaling	0.04080404
GO:0050804	modulation of chemical synaptic transmission	0.04542497
GO:0099177	regulation of trans-synaptic signaling	0.04733191
GO:0060291	long-term synaptic potentiation	0.04916304

Table 7 GO terms enriched in the top 10% of genes positively associated with cortical mean diffusivity in preHD

GO term	Description	P value
GO:0048856	anatomical structure development	1.54E-15
GO:0032502	developmental process	2.80E-15
GO:0009888	tissue development	1.91E-14
GO:0007275	multicellular organism development	1.96E-14
GO:0048731	system development	9.88E-14
GO:0032501	multicellular organismal process	3.54E-13
GO:0035295	tube development	4.26E-13
GO:0009653	anatomical structure morphogenesis	7.51E-13
GO:0048513	animal organ development	1.44E-12
GO:0007166	cell surface receptor signaling pathway	2.12E-10
GO:0072359	circulatory system development	6.14E-10
GO:0050896	response to stimulus	3.93E-09
GO:0001655	urogenital system development	7.86E-09
GO:0060429	epithelium development	8.92E-09
GO:0009887	animal organ morphogenesis	1.22E-08
GO:0048729	tissue morphogenesis	1.34E-08
GO:0035239	tube morphogenesis	1.59E-08
GO:0048869	cellular developmental process	8.25E-08
GO:0048646	anatomical structure formation involved in morphogenesis	8.64E-08
GO:0051716	cellular response to stimulus	1.05E-07
GO:0072001	renal system development	1.18E-07
GO:0030154	cell differentiation	1.32E-07
GO:0001568	blood vessel development	1.68E-07
GO:0007154	cell communication	1.77E-07
GO:0007165	signal transduction	3.53E-07
GO:0001822	kidney development	3.92E-07
GO:0023052	signaling	3.97E-07
GO:0001944	vasculature development	5.54E-07
GO:0030198	extracellular matrix organization	1.84E-06
GO:0003012	muscle system process	1.91E-06
GO:0043062	extracellular structure organization	1.95E-06
GO:0045229	external encapsulating structure organization	2.19E-06
GO:0050793	regulation of developmental process	3.51E-06
GO:0061061	muscle structure development	7.71E-06
GO:0042221	response to chemical	1.63E-05
GO:0051239	regulation of multicellular organismal process	1.66E-05
GO:0002009	morphogenesis of an epithelium	1.67E-05
GO:0048514	blood vessel morphogenesis	2.20E-05
GO:0048468	cell development	2.64E-05
GO:0008283	cell population proliferation	3.13E-05

GO:0003013	circulatory system process	3.19E-05
GO:0042127	regulation of cell population proliferation	5.79E-05
GO:0048598	embryonic morphogenesis	7.12E-05
GO:0007507	heart development	7.50E-05
GO:0006936	muscle contraction	8.38E-05
GO:0032835	glomerulus development	9.26E-05
GO:0072006	nephron development	9.28E-05
GO:0009790	embryo development	0.00012768
GO:0010646	regulation of cell communication	0.0001412
GO:0023051	regulation of signaling	0.00017321
GO:0060485	mesenchyme development	0.00018622
GO:0007155	cell adhesion	0.00020835
GO:0060537	muscle tissue development	0.00023428
GO:0040011	locomotion	0.00026759
GO:0050794	regulation of cellular process	0.00026773
GO:0048523	negative regulation of cellular process	0.00027189
GO:0016477	cell migration	0.00030187
GO:0048870	cell motility	0.00034446
GO:0051674	localization of cell	0.00034446
GO:0065007	biological regulation	0.00044841
GO:0050789	regulation of biological process	0.00051255
GO:0009966	regulation of signal transduction	0.00052275
GO:0001525	angiogenesis	0.00057047
GO:0070887	cellular response to chemical stimulus	0.00067304
GO:0008015	blood circulation	0.00106557
GO:0010033	response to organic substance	0.00122756
GO:0048583	regulation of response to stimulus	0.00123652
GO:0003008	system process	0.00148999
GO:0007229	integrin-mediated signaling pathway	0.00181874
GO:0051179	localization	0.00193381
GO:0003007	heart morphogenesis	0.00250094
GO:0030199	collagen fibril organization	0.0026993
GO:0048518	positive regulation of biological process	0.00271073
GO:0051240	positive regulation of multicellular organismal process	0.00275833
GO:0030334	regulation of cell migration	0.00276568
GO:0072073	kidney epithelium development	0.0031418
GO:0006928	movement of cell or subcellular component	0.00339396
GO:0072010	glomerular epithelium development	0.00381435
GO:0065008	regulation of biological quality	0.00389224
GO:2000145	regulation of cell motility	0.0040321
GO:0051094	positive regulation of developmental process	0.00413195
GO:0048762	mesenchymal cell differentiation	0.00507172
GO:0030855	epithelial cell differentiation	0.00553363

GO:0007399	nervous system development	0.00560258
GO:0009968	negative regulation of signal transduction	0.00627865
GO:2000026	regulation of multicellular organismal development	0.00736681
GO:0051270	regulation of cellular component movement	0.00818478
GO:0010648	negative regulation of cell communication	0.0082309
GO:0023057	negative regulation of signaling	0.00902192
GO:0048585	negative regulation of response to stimulus	0.00932869
GO:0048568	embryonic organ development	0.00972402
GO:0040012	regulation of locomotion	0.0109862
GO:0010604	positive regulation of macromolecule metabolic process	0.0109979
GO:0098609	cell-cell adhesion	0.01216966
GO:0072009	nephron epithelium development	0.01602324
GO:0032989	cellular component morphogenesis	0.01810838
GO:0014706	striated muscle tissue development	0.0190118
GO:0060562	epithelial tube morphogenesis	0.02034281
GO:0042592	homeostatic process	0.02420401
GO:0001704	formation of primary germ layer	0.02720685
GO:0071310	cellular response to organic substance	0.02762537
GO:0071560	cellular response to transforming growth factor beta stimulus	0.02935797
GO:0030030	cell projection organization	0.03114395
GO:0071363	cellular response to growth factor stimulus	0.03323373
GO:0009605	response to external stimulus	0.03475315
GO:0120036	plasma membrane bounded cell projection organization	0.03617613
GO:0035556	intracellular signal transduction	0.0367133
GO:0071559	response to transforming growth factor beta	0.0387638
GO:0048738	cardiac muscle tissue development	0.04269535
GO:0022603	regulation of anatomical structure morphogenesis	0.04573249
GO:0045595	regulation of cell differentiation	0.04629331
GO:0003015	heart process	0.04642439
GO:0009893	positive regulation of metabolic process	0.04878945

Table 8 GO terms enriched in the top 10% of genes negatively associated with cortical mean diffusivity in preHD

GO Term	Description	P value
GO:0045333	cellular respiration	1.26E-14
GO:0007005	mitochondrion organization	2.54E-14
GO:0042775	mitochondrial ATP synthesis coupled electron transport	1.93E-13
GO:0042773	ATP synthesis coupled electron transport	1.93E-13
GO:0015980	energy derivation by oxidation of organic compounds	4.21E-13
GO:0022904	respiratory electron transport chain	1.01E-12
GO:0009060	aerobic respiration	1.03E-12
GO:0019646	aerobic electron transport chain	1.64E-10
GO:0006119	oxidative phosphorylation	2.14E-10
GO:0022900	electron transport chain	3.81E-10
GO:0006091	generation of precursor metabolites and energy	2.18E-09
GO:0046034	ATP metabolic process	6.38E-07
GO:0006996	organelle organization	1.59E-05
GO:0006120	mitochondrial electron transport, NADH to ubiquinone	2.48E-05
GO:0006626	protein targeting to mitochondrion	5.64E-05
GO:0044260	cellular macromolecule metabolic process	9.04E-05
GO:0044267	cellular protein metabolic process	0.0001117
GO:0050794	regulation of cellular process	0.0001357
GO:0070585	protein localization to mitochondrion	0.00016914
GO:0019538	protein metabolic process	0.00017657
GO:1901564	organonitrogen compound metabolic process	0.00018942
GO:0072655	establishment of protein localization to mitochondrion	0.00040569
GO:0006839	mitochondrial transport	0.00062639
GO:0044248	cellular catabolic process	0.00071445
GO:0044257	cellular protein catabolic process	0.00112269
GO:0051603	proteolysis involved in cellular protein catabolic process	0.00218027
GO:0030163	protein catabolic process	0.0046225
GO:0009987	cellular process	0.00507333
GO:0009057	macromolecule catabolic process	0.00520806
GO:0007399	nervous system development	0.00592441
GO:0098662	inorganic cation transmembrane transport	0.00805982
GO:0044265	cellular macromolecule catabolic process	0.01069508
GO:0010257	NADH dehydrogenase complex assembly	0.01106478
GO:0032981	mitochondrial respiratory chain complex I assembly	0.01106478
GO:0022411	cellular component disassembly	0.01165835
GO:0098937	anterograde dendritic transport	0.01361016
GO:0009056	catabolic process	0.01593881
GO:0098655	cation transmembrane transport	0.01699261
GO:0033108	mitochondrial respiratory chain complex assembly	0.01876829
GO:1901565	organonitrogen compound catabolic process	0.02222081

GO:0098660	inorganic ion transmembrane transport	0.02463497
GO:0007275	multicellular organism development	0.02524858
GO:0043043	peptide biosynthetic process	0.0306083
GO:0048731	system development	0.03217434
GO:0033043	regulation of organelle organization	0.03999818
GO:0006511	ubiquitin-dependent protein catabolic process	0.04714753
GO:0006413	translational initiation	0.04913717

Table 9 List of genes involved in neurodevelopment

Gene
CYP26A1
TM6SF1
OSTN
TSHZ3
SSX2IP
FBXW7
SATB2
PART1
MCHR2
RORB

Figure 5 Principal component analysis of developmental genes expression in the AHBA

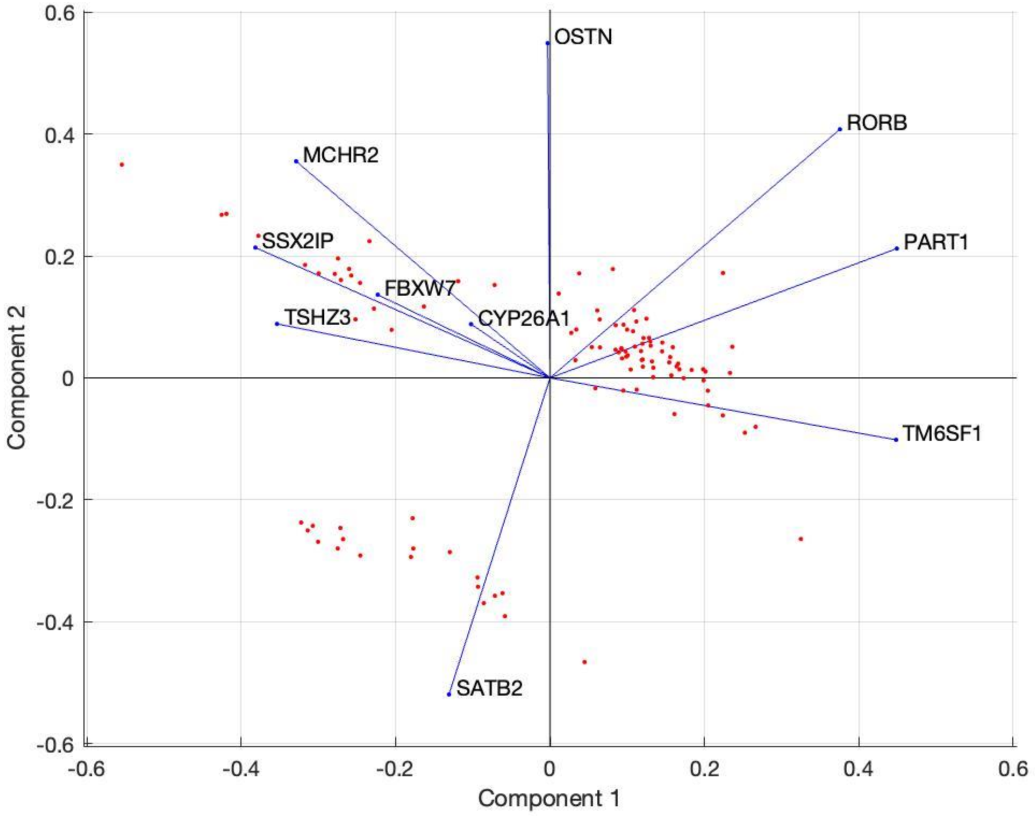

Table 10 EWCE analysis showing cell types enriched within 10%, 20% and 30% genes positively associated with cortical volume loss using the AIBS single-cell transcription and the DroNC datasets

AIBS				
10%				
**CellType**	**annotLevel**	**p**	**fold_change**	**sd_from_mean**
Astrocyte	1	0	1.353909108	8.299110199
Endothelial cell	1	0	1.516799804	9.576170262
GABAergic	1	1	0.738534785	-6.922914949
Glutamatergic	1	1	0.733600131	-6.578371223
Microglia	1	1	0.793471878	-4.19261074
Non-neuronal:Pericyte	1	0.75432	0.960598342	-0.691413515
Non-neuronal:VLMC	1	4.00E-05	1.279232786	4.13019496
Oligodendrocyte	1	1	0.721085153	-6.200361951
OPC	1	0.18284	1.036696015	0.898520698

DroNC				
10%				
**CellType**	**annotLevel**	**p**	**fold_change**	**sd_from_mean**
ASC	1	0	1.34645276	7.681571894
END	1	0	1.63841465	11.89567123
exCA	1	1	0.59021204	-13.32384462
exDG	1	1	0.63269777	-10.87461207
exPFC	1	1	0.50059859	-16.41763955
GABA	1	1	0.61327637	-11.19780563
MG	1	0.99708	0.80969043	-2.52350314
NSC	1	0	1.24142342	5.060028977
ODC	1	1	0.54955331	-7.749730421
OPC	1	0.60126	0.98525926	-0.284731918

AIBS				
20%				
**CellType**	**annotLevel**	**p**	**fold_change**	**sd_from_mean**
Astrocyte	1	0	1.387954424	13.04986428
Endothelial cell	1	0	1.42041461	11.22947896
GABAergic	1	1	0.804449293	-7.379729741
Glutamatergic	1	1	0.745769084	-8.921126631
Microglia	1	0.9908	0.920356912	-2.316692336
Non-neuronal:Pericyte	1	0.37204	1.012548585	0.31840569
Non-neuronal:VLMC	1	3.00E-05	1.189860721	4.037591027
Oligodendrocyte	1	1	0.757901865	-7.6506672

DroNC				
20%				
**CellType**	**annotLevel**	**p**	**fold_change**	**sd_from_mean**
ASC	1	0	1.39472975	12.59563621
END	1	0	1.51561549	13.85855906
exCA	1	1	0.66361888	-15.60647869
exDG	1	1	0.69890548	-12.6708484
exPFC	1	1	0.58189481	-19.59729137
GABA	1	1	0.69691564	-12.60090874
MG	1	0.97035	0.9039518	-1.828687166
NSC	1	0	1.20454417	6.140533943

OPC	1	0.00085	1.090628268	3.200649174

ODC	1	1	0.66635522	-8.198536756
OPC	1	0.00303	1.10283789	2.878642831

AIBS				
30%				
**CellType**	**annotLevel**	**p**	**fold_change**	**sd_from_mean**
Astrocyte	1	0	1.397611942	16.44271812
Endothelial cell	1	0	1.380912729	12.62480903
GABAergic	1	1	0.866094523	-6.238653073
Glutamatergic	1	1	0.806798537	-8.371260052
Microglia	1	0.92292	0.960600069	-1.416590753
Non-neuronal:Pericyte	1	0.50275	0.999591887	-0.012771994
Non-neuronal:VLMC	1	0	1.188903604	5.003602412
Oligodendrocyte	1	1	0.837830758	-6.373554494
OPC	1	1.00E-05	1.111423885	4.863674521

DroNC				
30%				
**CellType**	**annotLevel**	**p**	**fold_change**	**sd_from_mean**
ASC	1	0	1.37236281	14.67029365
END	1	0	1.45142747	15.0131092
exCA	1	1	0.74458737	-14.53057985
exDG	1	1	0.77876976	-11.49078209
exPFC	1	1	0.67553844	-18.55366635
GABA	1	1	0.76399753	-12.07368668
MG	1	0.90296	0.94604369	-1.284192224
NSC	1	0	1.26414476	9.796549827
ODC	1	1	0.74026644	-7.93996173
OPC	1	2.00E-05	1.12898849	4.471730526

Table 11 EWCE analysis showing cell types enriched within 10%, 20% and 30% genes negatively associated with cortical volume loss using the AIBS single-cell transcription and the DroNC datasets

AIBS				
10%				
**CellType**	**annotLevel**	**p**	**fold_change**	**sd_from_mean**
Astrocyte	1	1	0.751282621	-5.727462114
Endothelial cell	1	0.99989	0.814581303	-3.418780092
GABAergic	1	0	1.289338014	7.52885599
Glutamatergic	1	0	1.528554718	12.80936116
Microglia	1	0	1.39010846	7.758335161
Non-neuronal:Pericyte	1	0.69644	0.970273406	-0.525157437
Non-neuronal:VLMC	1	0.99705	0.820174552	-2.649009422
Oligodendrocyte	1	0.79633	0.962076806	-0.837768296
OPC	1	0.27986	1.022792276	0.569506242

DroNC				
10%				
**CellType**	**annotLevel**	**p**	**fold_change**	**sd_from_mean**
ASC	1	1	0.80027258	-4.43213217
END	1	0.99025	0.8784664	-2.249973205
exCA	1	0	1.33124654	10.64371563
exDG	1	0.00152	1.10149167	3.005049741
exPFC	1	0	1.4859348	15.72430639
GABA	1	0	1.26624524	7.683529119
MG	1	0	1.78593381	10.1903561
NSC	1	0.99978	0.83910096	-3.310031996
ODC	1	0.99818	0.84481744	-2.678040506
OPC	1	0.39886	1.01092488	0.223056891

AIBS				
20%				
**CellType**	**annotLevel**	**p**	**fold_change**	**sd_from_mean**
Astrocyte	1	1	0.830369495	-5.529675154
Endothelial cell	1	0.99573	0.901925372	-2.556392234
GABAergic	1	0	1.287058073	10.54751146
Glutamatergic	1	0	1.466576669	15.91557349
Microglia	1	0	1.26797107	7.626606066
Non-neuronal:Pericyte	1	0.04568	1.068445219	1.711651528
Non-neuronal:VLMC	1	0.99612	0.876675157	-2.58241197
Oligodendrocyte	1	0.08639	1.043813719	1.373156167
OPC	1	0.10712	1.035487535	1.251155892

DroNC				
20%				
**CellType**	**annotLevel**	**p**	**fold_change**	**sd_from_mean**
ASC	1	0.99999	0.87785247	-3.822395923
END	1	0.85363	0.96010037	-1.050318637
exCA	1	0	1.32770586	14.86269279
exDG	1	0	1.19127833	7.930293466
exPFC	1	0	1.4434611	20.16325051
GABA	1	0	1.28303969	11.51953786
MG	1	0	1.49316224	9.084451396
NSC	1	0.99899	0.8982038	-2.988945989
ODC	1	0.7016	0.97766114	-0.545631638

OPC	1	0.05555	1.05735533	1.627505857

AIBS				
30%				
**CellType**	**annotLevel**	**p**	**fold_change**	**sd_from_mean**
Astrocyte	1	1	0.862459871	-5.540648885
Endothelial cell	1	0.98857	0.93147825	-2.234802747
GABAergic	1	0	1.28999972	13.11684996
Glutamatergic	1	0	1.439123021	18.60834023
Microglia	1	0	1.25202809	8.817074202
Non-neuronal:Pericyte	1	4.00E-05	1.127297005	3.909686486
Non-neuronal:VLMC	1	0.85138	0.959930191	-1.035120335
Oligodendrocyte	1	0.00324	1.071824463	2.774391129
OPC	1	0.01531	1.050766218	2.212861758

DroNC				
30%				
**CellType**	**annotLevel**	**p**	**fold_change**	**sd_from_mean**
ASC	1	0.99966	0.91550453	-3.277818412
END	1	0.40793	1.00692061	0.226057812
exCA	1	0	1.35385033	19.5968077
exDG	1	0	1.25105204	12.79031289
exPFC	1	0	1.44168807	24.52795228
GABA	1	0	1.31477528	15.68834141
MG	1	0	1.41549192	9.56641154
NSC	1	0.96265	0.95129317	-1.755631442
ODC	1	0.2104	1.02650222	0.797334449
OPC	1	0.0033	1.0804842	2.825918634

Table 12 EWCE analysis showing cell types enriched within the top 10%, 20% and 30% genes positively associated with increases in cortical mean diffusivity using the AIBS single-cell transcription and the DroNC-seq datasets

AIBS				
10%				
**CellType**	**annotLevel**	**p**	**fold_change**	**sd_from_mean**
Astrocyte	1	0	1.35185271	5.965243887
Endothelial cell	1	0	1.383532471	5.15933219
GABAergic	1	1	0.764747329	-4.513687551
Glutamatergic	1	1	0.515968606	-8.687005258
Microglia	1	0.99841	0.812427303	-2.738085156
Non-neuronal:Pericyte	1	0	1.597978975	7.550388206
Non-neuronal:VLMC	1	1.00E-04	1.378132796	4.100850647
Oligodendrocyte	1	0.1977	1.052620864	0.842280753
OPC	1	0.99972	0.824903964	-3.133989023

DroNC				
10%				
**CellType**	**annotLevel**	**p**	**fold_change**	**sd_from_mean**
ASC	1	0	1.57645664	9.439619241
END	1	0	1.81610879	10.95365082
exCA	1	1	0.73069223	-6.486611145
exDG	1	1	0.76689478	-5.093094381
exPFC	1	1	0.53267438	-11.47162131
GABA	1	1	0.74671923	-5.419449608
MG	1	0.14552	1.10897559	1.057910119
NSC	1	0	1.55464919	8.412579734
ODC	1	0.00252	1.24558354	3.068885649
OPC	1	0.13744	1.07670855	1.095502269

AIBS				
20%				
**CellType**	**annotLevel**	**p**	**fold_change**	**sd_from_mean**
Astrocyte	1	0	1.383944686	9.196347063
Endothelial cell	1	0	1.435999097	8.421329612
GABAergic	1	1	0.699324611	-8.198624807
Glutamatergic	1	1	0.509830106	-12.34887586
Microglia	1	1	0.792791074	-4.346796512
Non-neuronal:Pericyte	1	0	1.368420156	6.636156394
Non-neuronal:VLMC	1	0	1.424895812	6.591154791
Oligodendrocyte	1	1.00E-05	1.202255847	4.623958163
OPC	1	0.86693	0.957015424	-1.112918905

DroNC				
20%				
**CellType**	**annotLevel**	**p**	**fold_change**	**sd_from_mean**
ASC	1	0	1.65282693	15.13185926
END	1	0	1.76780964	14.6942955
exCA	1	1	0.75641599	-8.356195585
exDG	1	1	0.79382443	-6.40723919
exPFC	1	1	0.56514223	-15.02838872
GABA	1	1	0.72455623	-8.456312731
MG	1	0.04684	1.12475163	1.741140073
NSC	1	0	1.56951732	12.32739126
ODC	1	0	1.56144376	10.00644032

OPC	1	0.00011	1.20309904	4.199103427

AIBS				
30%				
**CellType**	**annotLevel**	**p**	**fold_change**	**sd_from_mean**
Astrocyte	1	0	1.382850921	11.29501202
Endothelial cell	1	0	1.396159021	9.436970635
GABAergic	1	1	0.732107482	-9.009602607
Glutamatergic	1	1	0.555634931	-13.82158626
Microglia	1	1	0.84664604	-3.952896771
Non-neuronal:Pericyte	1	0	1.260689751	5.798482975
Non-neuronal:VLMC	1	0	1.322314901	6.191359495
Oligodendrocyte	1	0	1.291017373	8.201278822
OPC	1	0.31509	1.0149275	0.476262662

DroNC				
30%				
**CellType**	**annotLevel**	**p**	**fold_change**	**sd_from_mean**
ASC	1	0	1.61890895	17.63355203
END	1	0	1.63224827	14.92875228
exCA	1	1	0.7709615	-9.620361368
exDG	1	1	0.83657619	-6.229946588
exPFC	1	1	0.60183042	-16.84588449
GABA	1	1	0.78075725	-8.237305915
MG	1	0.00822	1.14725194	2.524457876
NSC	1	0	1.51140068	13.63013351
ODC	1	0	1.6814185	14.93824617
OPC	1	0	1.26664328	6.808969087

Table 13 EWCE analysis showing cell types enriched within the top 10%, 20% and 30% of genes negatively associated with increases in cortical mean diffusivity in the AIBS single-cell transcription and the DroNC-seq datasets

AIBS				
10%				
**CellType**	**annotLevel**	**p**	**fold_change**	**sd_from_mean**
GABAergic	1	0	1.291348408	9.489950106
Glutamatergic	1	0	1.484499996	14.78665385
Non-neuronal:Pericyte	1	0	1.219889303	4.889603259
Microglia	1	0.00172	1.119730932	3.021554323
OPC	1	0.46155	1.002562658	0.08015723
Non-neuronal:VLMC	1	0.97458	0.896361968	-1.916698458
Endothelial cell	1	0.99997	0.830357137	-3.967546737
Astrocyte	1	1	0.829573919	-4.893773863
Oligodendrocyte	1	1	0.85369899	-4.072890172

DroNC				
10%				
**CellType**	**annotLevel**	**p**	**fold_change**	**sd_from_mean**
ASC	1	1	0.819621187	-5.023391842
END	1	0.92599	0.938935174	-1.427431723
exCA	1	0	1.215025304	8.618050571
exDG	1	0.00011	1.101974239	3.727088388
exPFC	1	0	1.459022386	18.41862579
GABA	1	0	1.255920963	9.150635384
MG	1	0.00667	1.160439131	2.599697401
NSC	1	0.98769	0.914757235	-2.191907383
ODC	1	1	0.72571792	-5.866924314
OPC	1	0.86503	0.956083392	-1.098687253

AIBS				
20%				
**CellType**	**annotLevel**	**p**	**fold_change**	**sd_from_mean**
GABAergic	1	0	1.286047401	13.101303
Glutamatergic	1	0	1.441544817	18.95089857
Microglia	1	0	1.187823877	6.683467061
Non-neuronal:Pericyte	1	0	1.158757869	4.985545338
OPC	1	0.05471	1.036945062	1.617121386
Non-neuronal:VLMC	1	0.98864	0.914475142	-2.242078748
Oligodendrocyte	1	0.99568	0.933883017	-2.576048077
Endothelial cell	1	0.99901	0.908885166	-3.000791769
Astrocyte	1	1	0.868018044	-5.379917223

DroNC				
20%				
**CellType**	**annotLevel**	**p**	**fold_change**	**sd_from_mean**
ASC	1	1	0.84913928	-5.890438794
END	1	0.71124	0.982892451	-0.566039462
exCA	1	0	1.244824011	13.64126459
exDG	1	0	1.148588791	7.645248349
exPFC	1	0	1.417845641	23.49419983
GABA	1	0	1.26250556	13.19528713
MG	1	2.00E-05	1.19251754	4.461603984
NSC	1	0.93793	0.957920005	-1.531726174
ODC	1	1	0.806806676	-5.809776411
OPC	1	0.50095	0.999434024	-0.019834678

AIBS				
30%				
**CellType**	**annotLevel**	**p**	**fold_change**	**sd_from_mean**
GABAergic	1	0	1.286047401	13.101303
Glutamatergic	1	0	1.441544817	18.95089857
Microglia	1	0	1.187823877	6.683467061
Non-neuronal:Pericyte	1	0	1.158757869	4.985545338
OPC	1	0.05471	1.036945062	1.617121386
Non-neuronal:VLMC	1	0.98864	0.914475142	-2.242078748
Oligodendrocyte	1	0.99568	0.933883017	-2.576048077
Endothelial cell	1	0.99901	0.908885166	-3.000791769
Astrocyte	1	1	0.868018044	-5.379917223

DroNC				
30%				
**CellType**	**annotLevel**	**p**	**fold_change**	**sd_from_mean**
ASC	1	1	0.848994819	-5.90012364
END	1	0.71285	0.982844042	-0.56726714
exCA	1	0	1.24482382	13.63889306
exDG	1	0	1.148551837	7.621712438
exPFC	1	0	1.417915372	23.45320858
GABA	1	0	1.262678434	13.18277982
MG	1	3.00E-05	1.192544175	4.463107473
NSC	1	0.93874	0.957970694	-1.534560019
ODC	1	1	0.806811657	-5.867624034
OPC	1	0.49809	0.999504746	-0.017354254
